# Yap controls notochord formation and neural tube patterning by integrating mechanotransduction with *FoxA2* and *Shh* expression

**DOI:** 10.1126/sciadv.adf6927

**Published:** 2023-06-14

**Authors:** Caiqi Cheng, Qian Cong, Yuchen Liu, Yizhong Hu, Guoyan Liang, Kevin Marc Manquiquis Dioneda, Yingzi Yang

**Affiliations:** Department of Developmental Biology, Harvard School of Dental Medicine, Harvard Stem Cell Institute, 188 Longwood Ave., Boston, MA 02115, USA.

## Abstract

Correct notochord and neural tube (NT) formation is crucial to the development of the central nervous system and midline structures. Integrated biochemical and biophysical signaling controls embryonic growth and patterning; however, the underlying mechanisms remain poorly understood. Here, we took the opportunities of marked morphological changes during notochord and NT formation and identified both necessary and sufficient roles of Yap, a key mechanosensor and mechanotransducer, in biochemical signaling activation during formation of notochord and floor plate, the ventral signaling centers that pattern the dorsal-ventral axis of NT and the surrounding tissues. We showed that Yap activation by a gradient of mechanical stress and tissue stiffness in the notochord and ventral NT induces *FoxA2* and *Shh* expression. Hedgehog signaling activation rescued NT patterning defects caused by *Yap* deficiency, but not notochord formation. Therefore, mechanotransduction via Yap activation acts in feedforward mechanisms to induce *FoxA2* expression for notochord formation and activate *Shh* expression for floor plate induction by synergistically interacting with FoxA2.

## Teaser

Mechanotransduction triggers a biochemical signaling cascade via Yap activation to control neural tube and trunk development.

## INTRODUCTION

Formation of notochord—a central player in early vertebrate development—and morphogenesis of vertebrate neural tube (NT)—the embryonic precursor of the central nervous system (CNS)—have been studied since the early days of embryology. Notochord patterns NT and the surrounding tissues, including somites, by secreting Sonic Hedgehog (Shh) and gives rise to the nucleus pulposus, a critical component in the spine (*1*). NT defects (NTDs) are one of the most common birth defects in humans (*2*, *3*). Both genetic and environmental factors have been associated with NTDs (*4*), reflecting the interactive complexity of multiple contributing processes to NT morphogenesis. Despite the interventions to reduce NTDs by folic acid supplementation before and during gestation, a significant fraction of NTDs remains unpreventable and/or difficult to treat (*5*–*7*), which demands better understanding of cellular and molecular mechanisms governing NT development.

Complex networks of biochemical signaling have been found to direct notochord and NT development of higher vertebrate embryos. However, much less is known about the mechanisms underlying biophysical regulation of development. Embryonic cells constantly receive biophysical stimuli such as stress, strain, and fluid flow, which are generated by gravity, cell movement, and cell-cell or cell–extracellular matrix interactions, during which myriad forces are at play to regulate morphogenesis, representing the most underexplored frontier of developmental biology. Mechanotransduction, the conversion of mechanical forces into biological signals, is a fundamental regulatory scheme for revealing environmental features to almost all cells during embryonic development and sensory perception (*8*–*10*). However, the precise instructive role of mechanotransduction in cell fate determination and patterning has remained long neglected, in part because of the difficulties in translating the physical cues in molecular terms. Notochord formation involves extensive morphogenesis, such as convergent extension and apical constriction (*11*). The folding-based primary neurulation is intimately associated with notochord formation. Together, they provide systems to explore how physical forces are integrated with biochemical signaling pathways to regulate complex embryonic development. Primary neurulation is a complex process subjected to intrinsic and extrinsic forces, mechanical constraints from neighboring tissues, and the environment. Cellular processes, such as apical constriction and cell intercalation, are responsible for shaping the NT (*12*, *13*). At the molecular level, Yes-associated protein 1 (Yap) and its paralog, transcriptional coactivator with PDZ-binding motif (Taz), are found to mediate a broad range of mechanical cues and translate them into cell-specific transcriptional programs (*14*, *15*). Yap and Taz are coactivators that mediate the transcriptional regulation of Hippo signaling, an evolutionarily conserved serine/threonine kinase cascade that critically controls cell proliferation and survival (*16*, *17*). Before our work, Yap is thought to be gatekeepers of progenitor cell proliferation in several contexts during embryonic development (*18*–*20*). While *Taz* is not required for embryonic development (*21*), *Yap^−/−^* mouse embryos die very early [around embryonic day 8.5 (E8.5)] with severely retarded growth and disorganized embryonic tissues (*22*), precluding a clear mechanistic dissection of its precise roles in development.

Here, we found that dorsoventral (DV) patterning of the NT and surrounding tissues requires Yap activation, which reads a DV gradient of mechanical forces and promotes the expression of the ventralizing factor Shh in the ventral organizing centers: the notochord and floor plate (FP) of the NT (*23*–*25*). It is known that the transcription factor Forkhead box protein A2 (FoxA2) is expressed specifically in the notochord and FP, where it activates *Shh* expression (*26*–*30*). In the FP, FoxA2 is also activated by Shh signaling in FP (*31*, *32*), forming a positive feedback loop with Shh. We show here that Yap is both necessary and sufficient to control DV patterning by activating *FoxA2* expression in the notochord and synergistically interacting with FoxA2 to activate *Shh* expression in the FP. We therefore have identified a previously unknown instructive role of cell mechanics in setting up the ventral organizing centers by selectively activating Yap in the notochord and FP.

## RESULTS

### The developing NT exhibits a gradient of tissue stiffness along the DV axis

**Fig. 1.**
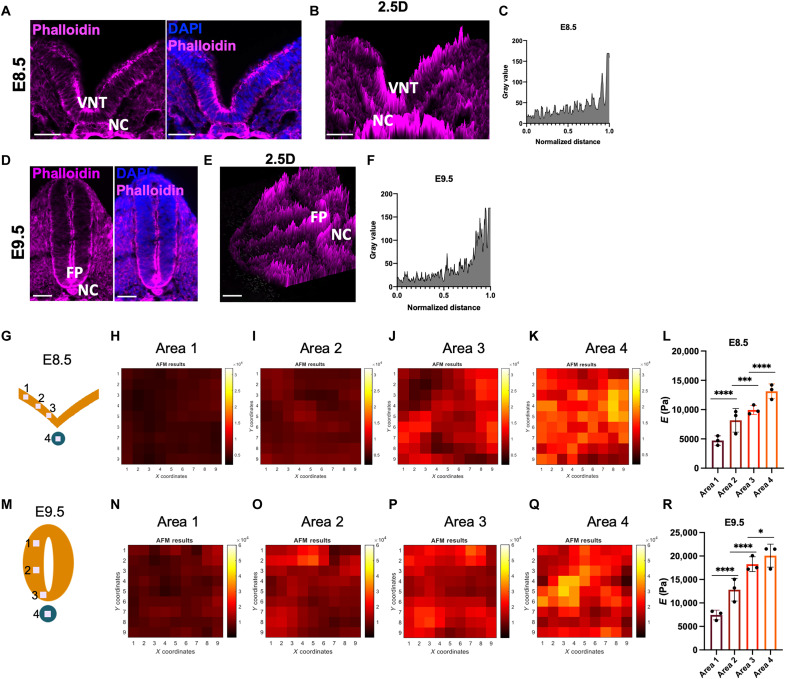
Ventral-dorsal stiffness gradient in the developing neural tube (NT). (**A**, **B**, **D**, and **E**) Representative images of three analyzed embryos of the same stage are shown. Phalloidin staining of filamentous actin (F-actin) in E8.5 (A and B) and E9.5 (D and E) neural plate. Scale bars, 20 μm. (B and E) 2.5D reconstruction of laser scanning image stacks of phalloidin staining. (**C** and **F**) Quantification of the phalloidin immunofluorescence intensity as the mean gray value in the NT of a representative embryo. The *x* axis represents distance, normalized to a scale of 0 to 1, from the dorsal domain (0) to the ventral domain (1.0). (**G** and **M**) Schematic of the experimental setup of E8.5 (G) and E9.5 (M). The atomic force microscopy (AFM) cantilever with a tip probe was navigated over neural plate using optical microscopy. The neural plate was shown in orange and the notochord in blue. Areas 1 to 3 in the NT and area 4 in the notochord were measured by AFM. For each area, 9 × 9 points were measured in a 14-μm by 14-μm (G) or 20-μm by 20-μm (M) region. Analyses were performed in three independent embryos of the same embryonic stage, and similar heatmap patterns were obtained. (**H** to **K** and **N** to **Q**) Young’s modulus *E* was calculated by fitting the force-indentation curves using a standard Hertz model, as means to measure surface stiffness. For each area in (G) and (M), the representative distribution of *E* within the area was shown in a heatmap using a customized MATLAB script. The average *E* values of the three analyzed embryos were plotted in (**L**) and (**R**). NC, notochord; VNT, ventral NT; FP, floor plate; DAPI, 4′,6-diamidino-2-phenylindole. *P < 0.05, ***P < 0.001, and ****P < 0.0001; one-way analysis of variance (ANOVA) followed by Tukey’s multiple comparisons tests.

Stress fiber [large bundle of actin filament (F-actin)] formation participates in surface stiffness sensing when cells adhered to a compliant surface (*33*–*35*). Our first attempt to determine the mechanical environment of the developing NT was to perform phalloidin staining to determine intracellular F-actin, which has been shown as both a tension sensor and force-generating mechanotransducer. We found that in the developing midline at E8.5 and E9.5, which are before and after NT closure, respectively, there was a ventral to dorsal gradient of F-actin abundance (Fig. 1, A to F). In the notochord, ventral NT, and FP, F-actin was most abundant. This is consistent with the observation that apical constriction in the midline of the neural plate drives NT formation (*36*) and the hypothesis that during notochord morphogenesis, apical constriction plays a role in its emergence from the mesoendodermal epithelium (*11*).

The DV gradient of F-actin distribution and its enrichment in the ventral midline cells (FP and notochord) led us to determine whether there is an associated gradient of tissue rigidity or stiffness. We therefore directly measured the rigidity of the developing NT by atomic force microscopy (AFM). We observed a DV gradient of tissue stiffness of the NT and notochord before (Fig. 1, G to L) and after (Fig. 1, M to R) NT closure at E8.5 and E9.5, respectively. Again, the highest rigidity was found in the notochord, ventral NT, and FP, where we found the strongest phalloidin staining. We also found that, at E8.5, there was a gradient of phospho-myosin light chain, which has been reported to contribute to stress fiber assembly and mechanics (*37*), with a stronger signal in the notochord and ventral NT (fig. S1). These results indicate that there is a DV gradient of tissue rigidity in the early developing NT, suggesting that the ventral NT cells may experience higher tension. Ventral tissues, including the notochord, exhibited stronger rigidity.

### Concomitant up-regulation of Yap protein levels and activity with Shh expression in the notochord and FP

**Fig. 2.**
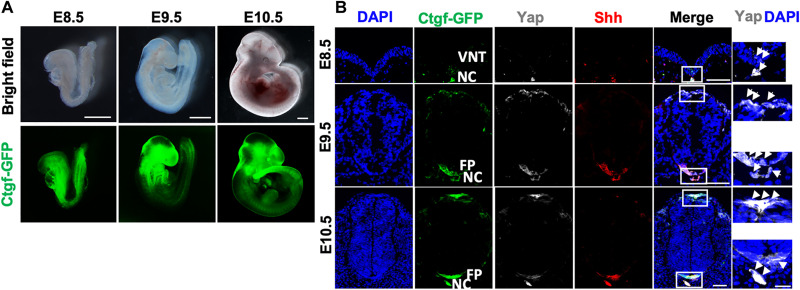
Yes-associated protein 1 (Yap) activation colocalized with Sonic Hedgehog (Shh) expression in the notochord and developing neural tube (NT). Images are representative of at least three embryos. (**A**) Whole-mount images of connective tissue growth factor–green fluorescent protein (*Ctgf-GFP*) embryos harvested at E8.5, E9.5, and E10.5. Ctgf-GFP^+^ cells were found in the NT, heart, brain, and limb buds. Scale bars, 500 μm. (**B**) Representative images of the cryosections double immunofluorescence–stained against GFP, Yap, and Shh. The trunks of E8.5, E9.5, and E10.5 embryos right posterior to the forelimb bud were cross-sectioned. Scale bars, 50 μm. Right: Confocal images of Yap with 4′,6-diamidino-2-phenylindole (DAPI); white arrows indicate Yap nuclear colocalization. Scale bar, 20 μm.

On the basis of our understanding of the mechanical forces involved in proper morphogenesis and the circumstances for Yap activation, we next asked whether our observed DV gradient of tissue rigidity was correlated with Yap activation. Connective tissue growth factor (*Ctgf*) is a transcription target of Yap, and the *Ctgf-GFP* mice have been developed to detect Yap activities (*38*). We found that Ctgf–green fluorescent protein (GFP) was expressed in the developing embryos (Fig. 2A). In the cross section of the embryonic trunk, Ctgf-GFP was detected in the notochord and ventral NT at E8.5 (Fig. 2B). At E9.5 and E10.5, high GFP levels were also detected in the notochord and FP, as well as the roof plate of the newly closed NT (Fig. 2B). Fluorescent immunohistochemistry (IHC) with anti-Yap and anti-Shh antibodies revealed that higher Yap protein levels were nicely colocalized with Ctgf-GFP and Shh expression (Fig. 2B). These results indicate that Yap is specifically activated in the notochord, the ventral NT, and later in the FP, where mechanical tension is higher (Fig. 1) and Shh is expressed.

**Fig. 3.**
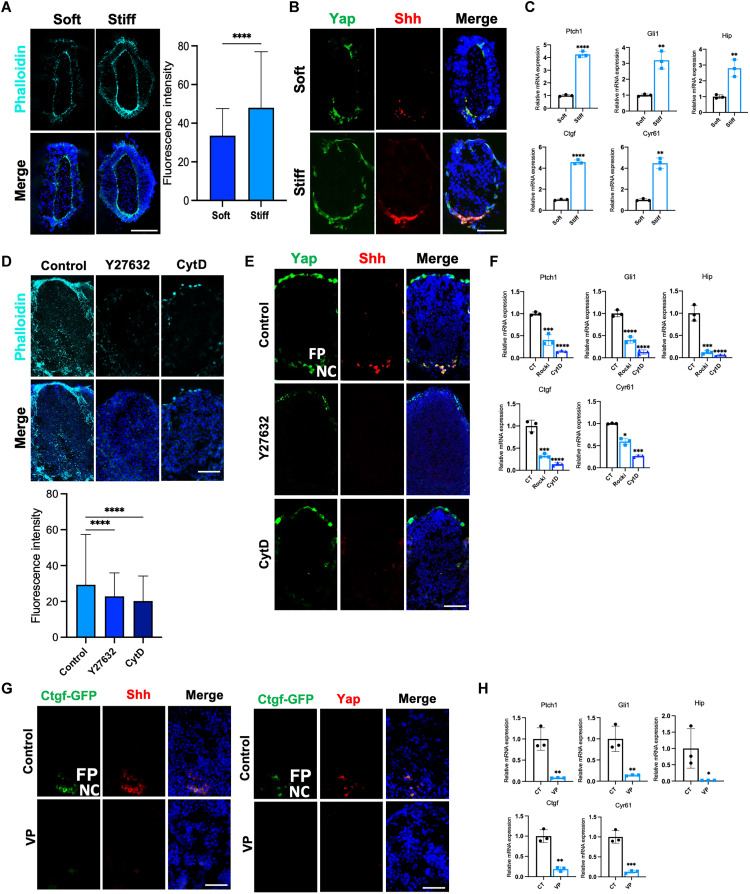
Alteration of matrix stiffness changes Yes-associated protein 1 (Yap) and Sonic Hedgehog (Shh) expression in the neural tube (NT) explant culture. (**A** to **C**) E8.5 neural plate explant embedded in soft and stiff collagen-alginate gel and cultured for 2 days. Representative images of immunofluorescent phalloidin staining (A) and double immunofluorescence staining against Yap and Shh (B). Scale bars, 50 μm. (C) Gene expression analyses by quantitative real-time polymerase chain reaction (qRT-PCR) of Hh signaling target genes *Ptch1*, *Gli1*, and *Hip* and Yap target genes *Ctgf* and *Cyr61* (means ± SD; *n* = 3 biological replicates). (**D** to **F**) E9.5 NT explant cultured in the F12 media for 2 days, with either vehicle or 5 μM Cyt D or 15 μM Y27632. Representative images of immunofluorescent Phalloidin staining (D) and double immunofluorescence staining against Yap and Shh (E). Scale bars, 50 μm. (F) Gene expression analyses by qRT-PCR of NT samples cultured in vehicle or 5 μM Cyt D or 15 μM Y27632 (means ± SD; *n* = 3 biological replicates). (**G** and **H**) E9.5 NT explant cultured in the F12 media for 24 hours, with either vehicle or 500 nM VP. Representative images of double immunofluorescence staining against green fluorescent protein (GFP)/Yap and GFP/Shh (G). Scale bars, 50 μm. (H) Gene expression analyses by qRT-PCR of NT samples cultured in vehicle or 500 nM VP (means ± SD; *n* = 3 biological replicates); CT, Control; *P < 0.05, **P < 0.01, ***P < 0.001, and ****P < 0.0001; one-way ANOVA followed by Tukey’s multiple comparisons tests.

To further test whether Yap activation requires stress fiber formation, we performed an embryonic explant culture (Fig. 3). The embryonic trunk explants were isolated from E8.5 embryos. The NT (with the somites removed) was cultured in collagen-alginate gel with different stiffness (Fig. 3, A to C) (*39*). A stiffer matrix resulted in more robust stress fiber formation (Fig. 3A) with stronger Yap activation and Shh expression (Fig. 3, B and C). We then treated the E9.5 whole trunk explants (between the forelimb and hind limb regions, with intact NT and somites) with a Rho kinase (Rock) inhibitor Y27632 or cytochalasin D (Cyt D) to suppress stress fiber formation by inhibiting actin polymerization. Both Y27632 and Cyt D greatly reduced F-actin formation (Fig. 3D), as well as Yap activation, Shh expression, and Shh signaling activities as assayed by expression levels of Hh signaling target genes Patched1 (Ptch1), GLI family zinc finger 1 (Gli1), and Hedgehog interacting protein 1 (Hhip1) (Fig. 3, E and F) (*40*–*42*). Furthermore, we treated the E9.5 whole trunk explants with the Yap/Tead inhibitor verteporfin (VP) (*43*) and found that such treatment abolished both Shh expression and Yap activation (Fig. 3, G and H). These results indicated that tissue stiffness and stress fiber formation led to Yap activation and Shh expression. They bring up an interesting possibility that mechanical forces generated during NT closure and maintenance of closed NT may regulate Shh expression through Yap activation.

### Yap is required for Shh and FoxA2 expression in regulating NT DV patterning

**Fig. 4.**
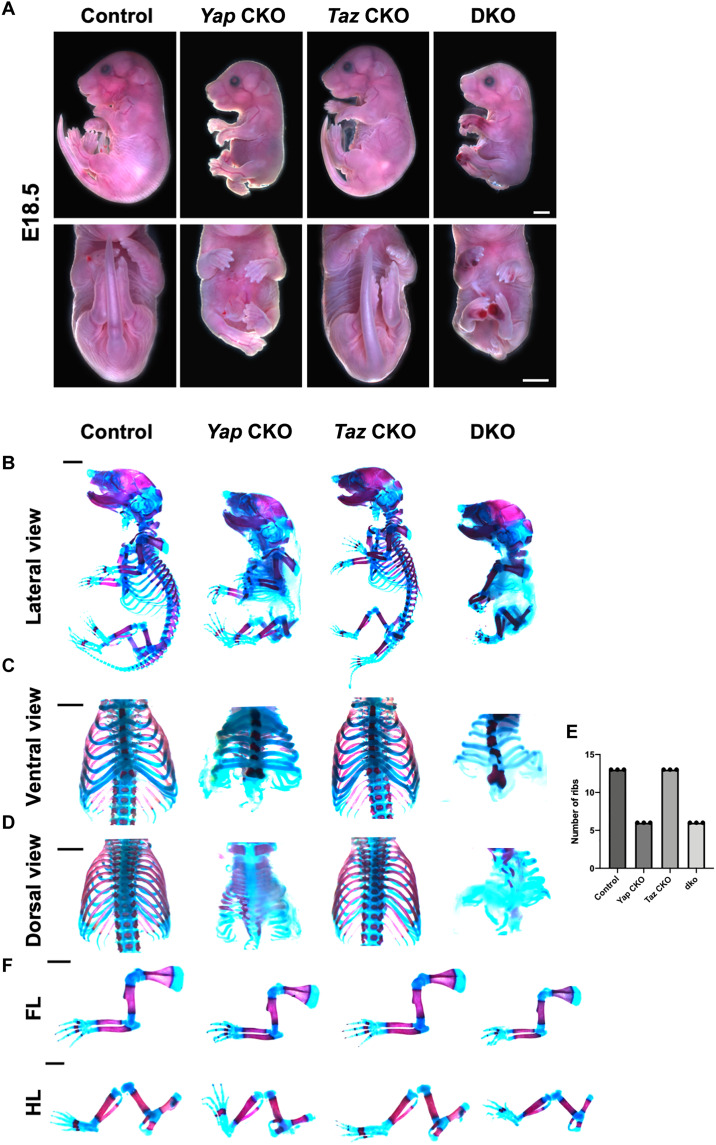
Loss of Yes-associated protein 1 (*Yap*)or *Yap/*transcriptional coactivator with PDZ-binding motif (*Taz*)in the Sonic Hedgehog (*Shh*)–expressing cells results in midline structure defects similar to those in the *Shh^−/−^* mutant embryos. (**A**) Whole-mount images of mouse embryos of indicated genotypes at E18.5. Scale bars, 0.5 cm. (**B**) Images of whole-mount Alizarin Red– and Alcian Blue–stained E18.5 embryos. (**C** and **D**) Images of Alizarin Red– and Alcian Blue–stained rib cages (ventral view and dorsal view) from embryos of the indicated genotypes at E18.5. Scale bars, 1 mm. (**E**) Quantification of rib number in embryos with the indicated genotypes. *n* = 3 in each genotype group. (**F**) Images of Alizarin Red– and Alcian Blue–stained forelimbs (FL) and hind limbs (HL) of the indicated genotypes at E18.5.

**Fig. 5.**
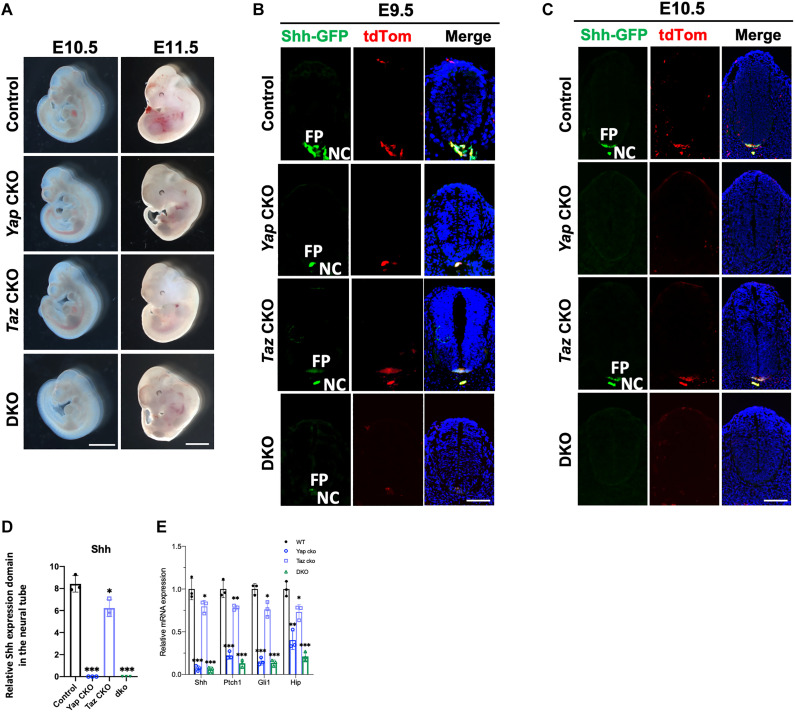
Yes-associated protein 1 (Yap) is required for Sonic Hedgehog (Shh) expression and formation of the notochord and floor plate (FP) in early embryonic development. (**A**) Whole-mount images of embryos (left lateral view) with indicated genotypes. Scale bars, 1 mm. (**B** and **C**) Representative immunofluorescent images of Shh–green fluorescent protein (GFP)and TdTomato (tdTom) in cross sections of neural tubes (NTs) of indicated genotypes. Scale bars, 50 μm (B) and 100 μm (C). (**D**) Quantification of the region with Shh-GFP^+^ cells along the dorsoventral (DV) axis as a percentage of the total DV length of the NT at E10.5 (fig. S5A) (C). (**E**) Quantitative real-time polymerase chain reaction (qRT-PCR) analyses of gene expression in E10.5 NT (means ± SD; *n* = 3 biological replicates). *P < 0.05, **P < 0.01, and ***P < 0.001; one-way ANOVA followed by Tukey’s multiple comparisons tests.

Shh, the ventralizing signal, is first expressed in the notochord at E7.5 and is induced in the FP by its expression in the notochord at E8.5 (*23*–*25*, *44*). As Yap was found to colocalize with Shh in the notochord and FP (Fig. 2B), we hypothesized that Yap may play a critical role activating *Shh* expression. To test this hypothesis, we used the *ShhCre* (*45*, *46*) to remove *Yap* and/or *Taz* in the notochord and FP (Fig. 4). We rationalized that if *Shh* expression requires *Yap* and/or *Taz*, then genomic deletion of *Yap/Taz* will lead to progressive loss of *Shh* expression as the deletion is irreversible even after *Shh-Cre* is inactivated. We found that the *Yap^f/f^;ShhCre* (*Yap* CKO) and the *Yap^f/f^;Taz^f/f^;ShhCre* (DKO) embryos were lethal at perinatal stages because of severe development defects of the lung, which is known to require Yap/Taz and Shh activities (*47*, *48*), as well as the skeleton such as the rib cages (Fig. 4, C and D). We therefore examined the E18.5 embryos (Fig. 4A). The *Yap* CKO embryos were smaller with shortened anterior-posterior (A-P) body axis, tail, and limbs. The phenotypes were more severe in the DKO embryos. The *Taz^f/f^;ShhCre* (*Taz* CKO) embryos were morphologically normal (Fig. 4A). The DKO embryos also showed a severe hemorrhage in the posterior limb (Fig. 4A), which is formed by *Shh*-expressing cells (*45*). We performed skeletal preparations and found that the DKO embryos almost completely copied the trunk phenotypes of the *Shh*^−/−^ embryos: lack of spinal column and most of the ribs (Fig. 4, B to E) (*31*). These phenotypes were also found in the *Yap* CKO embryos, although slightly less severe (Fig. 4, B to E). The *Yap* CKO and DKO embryos did not show cyclopia or a reduction in digit number, although posterior digit growth and ossification were reduced (Fig. 4F). Shortening of the A-P body axis and the tail in the *Yap* CKO and DKO embryos were obvious starting from E11.5, while the *Taz* CKO embryos appeared to be similar to the wild-type controls (Fig. 5A). Therefore, while Hippo signaling acts in other parts of the developing NT, in the *Shh*-expressing cells (*49*, *50*), we found that *Yap*, but not *Taz*, is required for development of midline structures, although loss of *Taz* further enhanced the defects caused by *Yap* loss. Hh and Hippo signaling exhibit both overlapping and distinct functions in embryonic development.

To test whether the midline phenotypes in the *Yap* CKO and *Yap*/*Taz* DKO embryos were caused by disruption in Shh expression, we cross-sectioned the trunk at the forelimb bud level (somite #5 to #11) from E9.5 and E10.5 embryos, followed by immunofluorescent IHC (Fig. 5, B and C). TdTomato (tdTom) expression from the *Rosa26* locus (*51*) was included in the breeding to trace the *Shh*-expressing and Cre-active cells. We found that Shh and the number of Shh-tdTom^+^ cells were greatly reduced in the notochord and FP in both the *Yap* CKO and DKO embryos, with more reduction observed in the DKO embryo at E9.5 (Fig. 5B). By E10.5, Shh and Shh-tdTom^+^ cells were not detectable in the *Yap* CKO and DKO embryos, while only slight reduction was found in the *Taz* CKO embryos (Fig. 5, C and D). These results indicate that *Yap* loss led to reduction of Shh expression at E9.5 first, followed by disappearance of *Shh*-expressing cells at E10.5. We should mention here that the *ShhCre* is a “knock-in” allele (*45*) and 50% of *Shh* expression was lost in the *ShhCre* mice. Therefore, the *Yap* CKO, *Taz* CKO, and DKO embryos were in a sensitized background of reduced *Shh*, which may have contributed to the strong *Yap* CKO and DKO phenotypes. We then determined Hh signaling activity in the E10.5 embryonic trunk by examining the expression of Hh signaling target genes *Ptch1*, *Gli1*, and *Hhip1* using quantitative real-time polymerase chain reaction (qRT-PCR) analysis, and found that Hh signaling was greatly reduced by *Yap* loss. Loss of *Taz* alone slightly reduced Hh signaling (Fig. 5E). Hematoxylin and eosin staining of the trunk sections indicated that, at E10.5, the NT was smaller along the DV axis in the *Yap* CKO embryo and smallest in the DKO embryo (fig. S2). In addition, the notochord was missing in both the *Yap* CKO embryo and DKO embryos (fig. S2). These results show that *Yap*, but not *Taz* alone, is required for the maintenance of Shh expression, as well as notochord and FP.

To test whether mild limb defects were due to late *Yap* removal using the *ShhCre*, we used the *Prrx1Cre* (*52*), which is expressed in the forelimb bud before *Shh* expression, to remove *Yap* and/or *Taz* (fig. S3A). In the *Prrx1Cre*-driven *Yap* CKO and *Yap/Taz* DKO embryos, severe hemorrhaging was observed in the midbrain at E10.5. By E12.5, the DKO embryos were dead, and the *Prrx1Cre Yap* CKO was unhealthy (fig. S3A). However, *Shh* expression, as assayed by whole-mount in situ hybridization, was only partially reduced in the DKO, while no significant reduction was observed in the *Yap* or *Taz* CKO (fig. S3B). These results suggest that the phenotypic variance in different tissues is likely due to the tissue-specific requirement for *Yap* in *Shh* expression. Different enhancers have been found to drive tissue-specific *Shh* expression (*28*, *30*). We therefore focused our analyses on the development of midline structures in the trunk where the phenotypes were most severe.

### Hedgehog signaling activation rescued the NT phenotypes of the *ShhCre;Yap^f/f^;Taz^f/f^* embryos

**Fig. 6.**
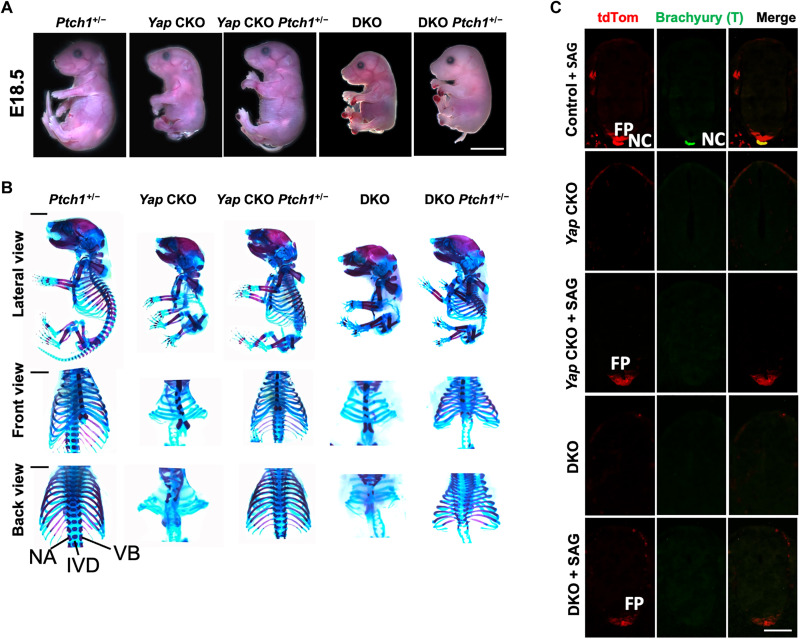
Sonic Hedgehog (Shh) signaling activation rescued the neural tube (NT), but not notochord phenotypes, caused by loss of Yes-associated protein 1 (*Yap*)or *Yap/ *transcriptional coactivator with PDZ-binding motif (*Taz*). (**A**) Representative whole-mount images of mouse embryos at E18.5. Scale bar, 1 cm. (**B**) Representative Alizarin Red and Alcian Blue staining of the indicated E18.5 whole embryos, the spine, and the rib cages (ventral view and dorsal view). Scale bars, 1 mm. NA, neural arch; VB, vertebral body. (**C**) Representative immunofluorescent images of TdTomato (tdTom) and brachyury (T) at E10.5 in trunk cross sections of indicated genotypes with or without small molecule agonist (SAG) treatment. Scale bar, 100 μm. (Mean ± SD; *n* = 3 biological replicates.)

Our finding that *Yap* was required for expression of Shh, a key ventralizing signal in development, led us to hypothesize that Hh signaling mediates critical aspects of the Yap activities in NT DV patterning. We therefore tested whether Hh signaling activation can rescue the phenotypes of *Yap* CKO and DKO. As Ptch1 inhibits Hh signaling (*53*), *Ptch1^+/−^* mice show elevated Hh signaling. We generated the *Yap^f/f^;Ptch1^+/−^;ShhCre* and the *Yap^f/f^;Taz^f/f^;Ptch1^+/−^;ShhCre* mouse embryos (Fig. 6, A and B). While the *Ptch1^+/−^* embryo was grossly normal as shown previously (Fig. 6A), the trunk phenotypes in the *Yap* CKO and DKO embryos were largely rescued by *Ptch1^+/−^* (Fig. 6, A and B). The tail in the *Yap^f/f^;Ptch1^+/−^;ShhCre* embryos was much longer than that in the *Yap* CKO embryo, although still shorter compared to that in the *Ptch1^+/−^* control (Fig. 6A). Skeletal preparation showed that increase of Hh signaling by removing one *Ptch1* copy restored formation and numbers of the rib and spinal column (Fig. 6B). However, the axial skeleton, particularly the vertebral body and the intervertebral discs (IVD), was still abnormal (Fig. 6B), suggesting that part of the Yap functions in development may be independent of Hh signaling.

We then asked whether stronger pharmacological activation of Hh signaling could better rescue the phenotypes of the *Yap* CKO and DKO embryos. It is known that SAG, a small molecule agonist of Hh signaling, directly binds to the SHH signaling transducer Smoothened (Smo) to activate it (*54*, *55*). SAG is able to cross the placenta, gut, and blood-brain barrier (*55*). We injected SAG intraperitoneally into pregnant mice (20 mg/kg) and found that up-regulating the compromised Shh signaling in the *Yap* CKO and DKO mutant embryos by SAG treatment rescued the axial skeleton and rib formation, although the tail was still thin and short (fig. S4, A and B). The IVD and vertebral bodies were abnormal, further indicating that Shh signaling activation in the absence of Yap activity may not be sufficient to promote normal IVD and vertebral body development.

As part of the IVD is derived from notochord and signals from notochord regulate vertebral body development, we examined notochord development and NT patterning by fluorescent IHC. At E10.5, notochord was marked by brachyury (T) IHC (*56*), and Shh-tdTom expression was found in both notochord and FP. Both notochord and FP were missing in the *Yap* CKO and DKO mutant embryos. SAG treatment largely restored Shh-tdTom^+^ cells in the FP, but not the notochord, as SAG treatment did not restore notochord formation (Fig. 6C). SAG treatment of the wild-type control embryos did not change T and Shh-tdTom tissue distribution (Fig. 6C). These results indicate that Yap is differentially required for notochord and FP development. Abnormal IVD in the mutant embryos rescued by *Ptch1^+/−^* or SAG treatment was due to failure to rescue the notochord formation, during which *Yap* is required independently of Shh expression and signaling.

### Yap controls NT DV patterning via promoting Shh expression

**Fig. 7.**
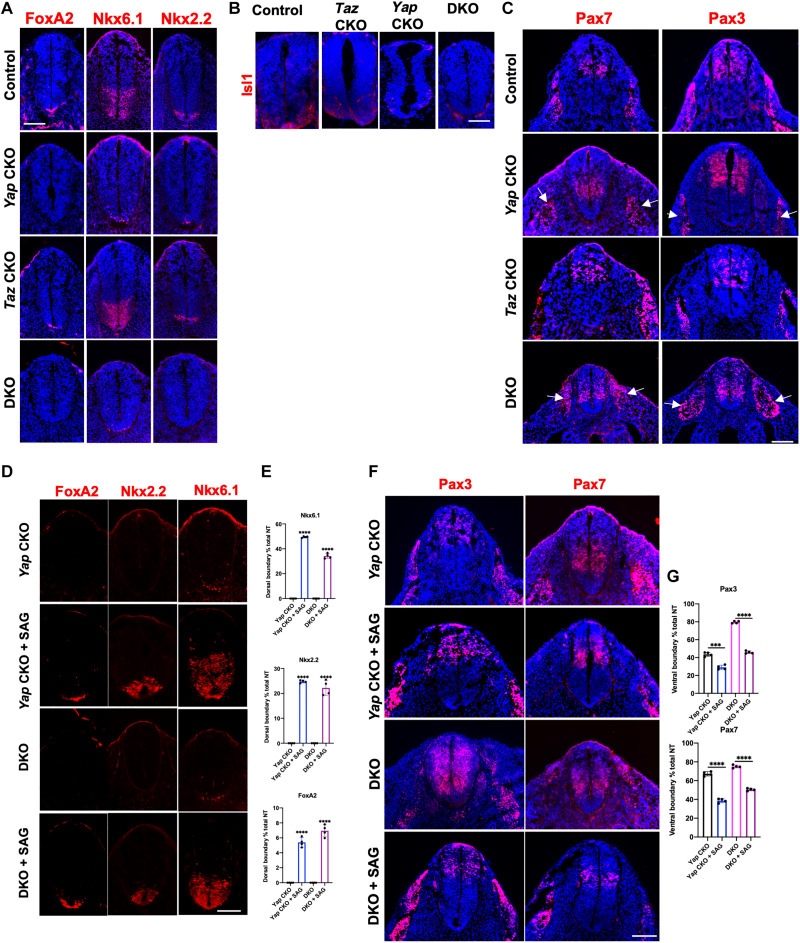
Yes-associated protein 1 (Yap) is required for dorsoventral (DV) patterning of the mouse neural tube (NT) by regulating Sonic Hedgehog (Shh) signaling. (**A**) Representative immunofluorescent images of FoxA2, Nkx6.1, and Nkx2.2 at E10.5 in NT of indicated genotypes. Scale bar, 100 μm. (**B** and **C**) Representative immunofluorescent images of motoneuron marker Islet1 (G) and Pax7 and Pax3 (H) at E10.5 in NT of indicated genotypes. Scale bars, 100 μm. (**D**) Representative immunofluorescent images of FoxA2, Nkx6.1, and Nkx2.2 at E10.5 in NT of indicated genotypes. Scale bar, 100 μm. (**E**) Quantitation of dorsal boundary of Nkx6.1, Nkx2.2, and FoxA2 gene expression as a percentage of total NT length. (**F**) Representative immunofluorescent images of Pax3 and Pax7 at E10.5 in NT of indicated genotypes. Scale bar, 100 μm. (**G**) Quantitation of ventral boundary of Pax3 and Pax7 gene expression as a percentage of total NT length (means ± SD; *n* = 3 biological replicates). ***P < 0.001 and ****P < 0.0001; one-way ANOVA followed by Tukey’s multiple comparisons tests.

As Shh signaling controls DV patterning of the developing NT, we next examined the expression of ventral NT markers by fluorescent IHC. It is known that Hh signaling regulates FoxA2 expression in the FP, NK2 Homeobox 2 (Nkx2.2), and NK6 Homeobox 1 (Nkx6.1) expression in the ventral NT (*57*, *58*). In the developing NT of E10.5 mouse embryos, we found loss of FoxA2 expression and markedly reduced Nkx2.2 and Nkx6.1 expression domains in the *Yap* CKO (Fig. 7A and fig. S5A). In the DKO embryo, neither FoxA2 nor Nkx2.2 was detected (Fig. 7A). However, in the midbrain sections of DKO embryos at E10.5, mild reduction—not absence of Shh, FoxA2, and NKx2.2—was found (fig. S3C), consistent with lack of obvious brain phenotypes in the DKO mutant. As Shh also induces motoneurons, we found that expression of ISL LIM Homeobox 1 (Islet1), a motoneuron marker (*23*, *25*, *59*), was also missing in DKO and greatly reduced in *Yap* CKO embryos (Fig. 7B). Consistently, the expression domains of dorsal NT markers Pax3 and Pax7 (*31*) were both expanded ventrally in the *Yap* CKO and DKO embryos (Fig. 7C and fig. S5B). Furthermore, expression of Paired box 3 (Pax3) and Pax7 in dermomyotome, the dorsal somite compartment, was found to be shifted ventrally in the *Yap* CKO and DKO embryos (Fig. 7B, arrows). These results show that removing *Yap* using *Shh-Cre* resulted in ventralized NT and somite due to loss of notochord and FP. Therefore, the *Yap* CKO and *Yap/Taz* DKO embryos phenocopied the *Shh^−/−^* mutant embryos in the trunk (*31*). SAG treatment rescued the expression patterns of ventral NT markers (FoxA2, Nkx2.2, and Nkx6.1) and dorsal markers (Pax3 and Pax7) in *Yap* CKO and DKO embryos (Fig. 7, D to G). Therefore, *Yap* is required for NT DV patterning by controlling Shh expression in the FP.

### Yap activation is sufficient to promote Shh and FoxA2 expression in the developing NT

**Fig. 8.**
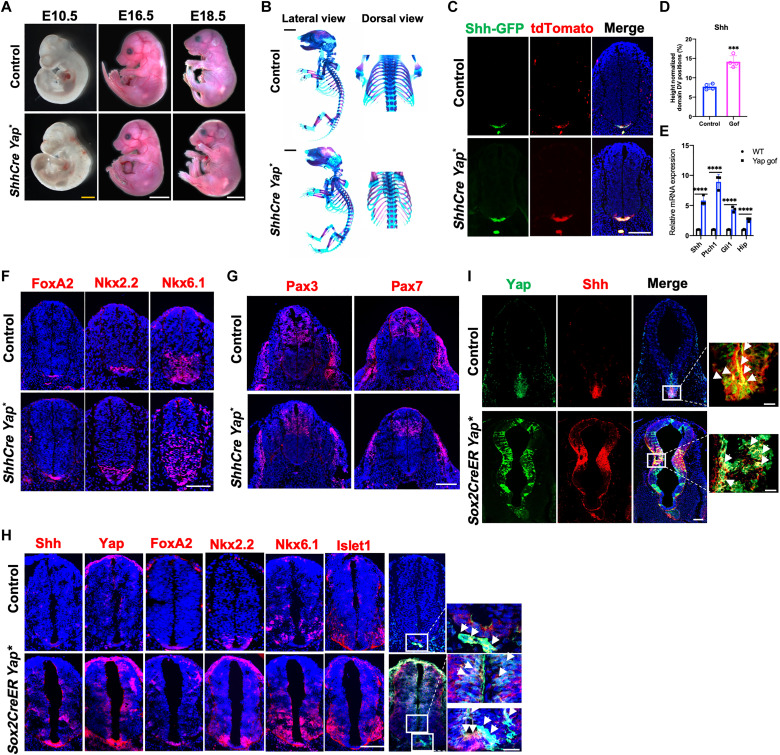
Yes-associated protein 1 (Yap) activation induced Sonic Hedgehog (Shh) and FoxA2 expression in the neural tube (NT). (**A**) Representative whole-mount images of mouse embryos at E10.5, E16.5, and E18.5. Scale bars, 500 μm (yellow) and 1 cm (white). (**B**) Representative whole-mount Alizarin Red and Alcian Blue staining of E18.5 embryos. The right panel showed the dorsal view of the ribcages. Scale bars, 1 mm. (**C**) Representative immunofluorescent images of Shh–green fluorescent protein (GFP) and TdTomato (tdTom) at E10.5 in NT of indicated genotypes. Scale bar, 100 μm. (**D**) Quantification of the Shh-GFP region along the DV axis as a percentage of the total DV length of the NT at E10.5. (**E**) Quantitative real-time polymerase chain reaction (qRT-PCR) analysis of gene expression in NT at E10.5 (means ± SD; *n* = 3 biological replicates). (**F**) Representative immunofluorescent images of Nkx6.1, Nkx2.2, and FoxA2 at E10.5 in NT of indicated genotypes. Scale bar, 100 μm. (**G**) Representative immunofluorescent images of Pax3 and Pax7 at E10.5 in NT of indicated genotypes. Scale bar, 100 μm. (**H**) Representative immunofluorescent images of Yap, Shh, FoxA2, Nkx2.2, Nkx6.1, and Islet1 at E10.5 in NT of indicated genotypes. Scale bar, 100 μm. Right: Confocal images show Yap with Shh; white arrows indicate Yap and Shh colocalization. Scale bar, 20 μm. (**I**) Representative immunofluorescent images of Yap and Shh at E10.5 in forebrain of indicated genotypes. Scale bar, 200 μm. Right: Confocal images show Yap with Shh; white arrows indicate Yap and Shh colocalization. Scale bars, 20 μm; Gof, Gain of function; (Mean ± SD; *n* = 3 biological replicates.) ***P < 0.001 and ****P < 0.0001; one-way ANOVA followed by Tukey’s multiple comparisons tests.

We then asked whether Yap activation in the developing NT is sufficient to promote Shh expression. The TetO-Yap* mice that allow Cre- and doxycycline (Dox)–inducible expression of an activated Yap (Yap*) (*38*) were used to activate Yap in the *Shh*-expressing cells using the *ShhCre* (Fig. 8). Pregnant female mice were fed with Dox water at E8.5. We found that Yap* expression did not grossly change the morphology of the embryos and axial skeleton, except that the tail was kinked (Fig. 8, A and B). Molecular analysis showed that Shh expression domain expanded (Fig. 8C). Enhanced *Shh* expression and Hh signaling activities were determined by qRT-PCR analyses of *Shh* and Hh signaling target gene expression (Fig. 8, D and E). Correlated with this, the NT was taller along the DV axis (fig. S6A), and the expression domains of FP and ventral NT markers (FoxA2, Nkx2.2, and Nkx6.1) (Fig. 8F and fig. S6B) expanded at the expense of dorsal markers Pax3 and Pax7 (Fig. 8G and fig. S6C).

We then asked whether Yap activation outside of the endogenous Shh expression domain is sufficient to induce ectopic Shh expression in the developing NT. We crossed the TetO-Yap* mice with the *Sox2CreER* mice (*60*) and induced Yap activation by tamoxifen (TM) injection and Dox water feeding at E7.5 (fig. S6E). We found that early Yap activation led to ectopic Shh expression in the NT outside of the endogenous Shh domain in both the trunk and the forebrain (Fig. 8, H and I). In addition, consistent with ectopic Shh expression induced by Yap activation in the NT, ectopic expression of FoxA2, Nkx2.2, and Nkx6.1 as well as the motoneuron maker Islet1 were found (Fig. 8H). These results demonstrate that Yap activation is sufficient to induce Shh expression in the NT. As Shh promotes cell proliferation and survival apart from patterning the DV axis of the NT, we examined cell proliferation and death in the developing NT at E10.5 (fig. S7). We found that, in the NT from *Yap* CKO and DKO embryos, cell proliferation, indicated by IHC of Ki67 or phospho-histone H3, was reduced throughout the NT (fig. S7, A to C), while cell death was increased compared to the wild-type controls. Conversely, in the mouse embryos with Yap activation (fig. S7, F to H), cell proliferation was increased throughout the NT and cell death was reduced.

### Yap promotes *Shh* expression via a feedforward mechanism by inducing *FoxA2* expression

**Fig. 9.**
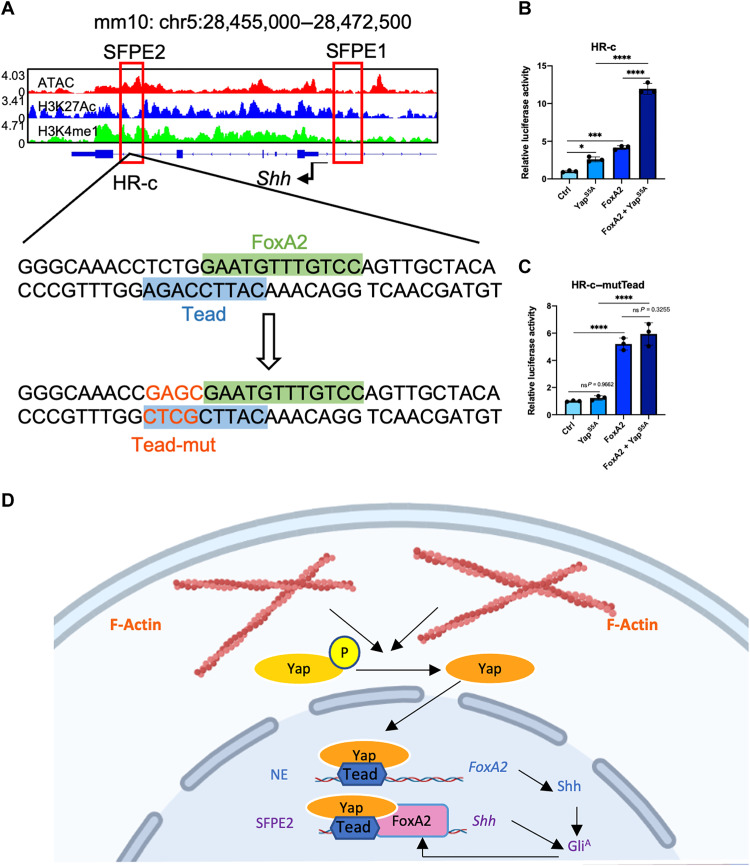
Sonic Hedgehog (*Shh*)expression is promoted by synergistic interaction between Yes-associated protein 1 (Yap)/transcriptional coactivator with PDZ-binding motif (Taz)and FoxA2. (**A**) Assay for transposase-accessible chromatin with sequencing (ATAC-seq) signal tracks of the *Shh* gene locus. Red box: Genomic regions of the SFPE2 and SFPE1. The HR-c region in SFPE2 was enlarged with FoxA2 and Tead binding sequences indicated. Mutated Tead binding site (TBS) was shown in orange. (**B** and **C**) Luciferase activity assay in 293T cells. The luciferase reporters under HR-c enhancer control were transfected together with the *PCMV-Flag-FoxA2* plasmid (*FoxA2*), the *PCMV*-*Flag-YAP^S5A^* plasmid (*YAP^S5A^*), or both. *P < 0.05, ***P < 0.001, and ****P < 0.0001; ns, no significant difference; one-way ANOVA followed by Tukey’s multiple comparisons tests. (**D**) Proposed model for the differential activity of Yap in regulating *FoxA2* expression in the notochord and *Shh* expression in the floor plate (FP). Yap is activated by stress fibers (F-actin) generated during notochord and neural tube (NT) morphogenesis. In the notochord, Yap binds to Tead1/2 on the notochord enhancer (NE) to promote *FoxA2* expression, which in turn activates *Shh* expression. In the FP, Yap binds to Tead factors and synergistically interacts with FoxA2 to induce *Shh* expression via the SFPE2 enhancer. Shh from the notochord and later FP signal through activating Gli transcription factors (Gli^A^) to activate *FoxA2* expression in the FP, forming a feedback loop with *Shh* that mediates the feedforward activities of Yap.

FoxA2 has previously been shown to control notochord and FP development (*61*, *62*). In previous efforts to identify the transcription regulation of *Shh* expression in development, consensus binding sites for FoxA2 class transcriptional regulators were identified in some *Shh* enhancers (*28*). *FoxA2* expression in NT is sufficient for promoting *Shh* expression (*29*, *30*, *63*). *FoxA2* expression is also activated by Shh signaling in the FP (*29*, *58*). As *Yap* is both necessary and sufficient for *Shh* expression in the NT (Figs. 5 and 8), we next asked whether Yap activates *Shh* expression by interacting with FoxA2 and therefore potentiating FoxA2 activity in the FP. We analyzed the Tead binding site (TBS), which mediate Yap/Taz transcription activities, in the previously identified Shh FP enhancer (SFPE) (Fig. 9A) (*28*). SFPE2 mediates FoxA2-dependent *Shh* regulation (*28*), and we identified a TBS close to and overlapping with the FoxA family DNA binding site in the core enhancer element HR-c of SFPE2 (Fig. 9A) (*30*), suggesting that FoxA2 and Yap/Tead may co-regulate *Shh* expression. To test this hypothesis, we performed luciferase assays of HR-c to determine transcriptional interaction of Yap and FoxA2 (Fig. 9, B and C). We found that, while activated Yap (Yap^S5A^) (*17*) or FoxA2 promoted HR-c luciferase activities, coexpression of both Yap^S5A^ and FoxA2 markedly increased HR-c luciferase activities, suggesting that the interaction is synergistic (Fig. 9B). Furthermore, we tested whether such an interaction depends on TBS by mutating it (Fig. 9C). Yap^S5A^ could not initiate activities of luciferase driven by TBS mutant HR-c, but FoxA2 still can. In addition, Yap^S5A^ could not further up-regulate FoxA2-mediated TBS mutant HR-c luciferase activity (Fig. 9C). These results suggest that Yap and FoxA2 interact synergistically to promote *Shh* expression in the FP via SFPE2. *FoxA2* itself is a critical target gene of Tead1 and Tead2 during mesoderm development including notochord formation (*64*). Our data that Yap was required for *FoxA2* expression and notochord formation further indicate that Yap activity in these processes is likely mediated by Tead1 and Tead2 via Teads binding elements (TBEs), and *FoxA2* expression, in turn, activates *Shh* expression (Fig. 9D). We therefore propose a model that Yap activated by mechanical stimuli is required for notochord formation and FP induction, likely via two feedforward routes. Yap promotes *FoxA2* expression in the notochord and *Shh* expression in the FP. In the FP, our data suggest that Yap binds to the TBE of SFPE2 to synergize with FoxA2, which is activated by Shh produced from the notochord or later from FP itself via activated Gli transcription factors (Gli^A^), for induction of *Shh* expression (Fig. 9D).

## DISCUSSION

Here, we provide evidence that growth and patterning in embryonic development can be regulated by mechanical stimuli generated during morphogenesis. We show that the forming notochord and NT exhibit a stiffness gradient along the DV axis, which likely results in localized activation of Yap transcription factor, a mediator of mechanotransduction, in the ventral midline signaling centers: notochord and FP (Fig. 9D). We further show that *Yap* is required to induce *FoxA2* expression and, thus, *Shh* expression in the notochord. In the FP, Yap is both necessary and sufficient to promote *Shh* expression in the NT by interacting with FoxA2 (Fig. 9D). While Hh signaling mediates Yap activities in NT patterning, Yap is required for notochord formation independently of Hh signaling.

Extensive progress has been made in deciphering the pathway downstream of Shh, but little is known about the mechanisms involved in activating *Shh* gene expression in discrete regions. The establishment of regional identity along the two major axes (A-P and DV) of the vertebrate nervous system and the trunk involves the coordinated expression of signaling molecules localized in time and space to discrete organizing centers (*65*, *66*). Two of such embryonic territories, the axial mesoderm (prechordal plate and notochord) underlying the neural plate and the ventral midline of the spinal cord (FP), have been defined as organizing centers based on their ability to regulate growth and patterning of the CNS and the surrounding tissues such as the somite (*65*, *67*, *68*). The critical role of *Shh* in the signaling centers has been revealed by extensive studies of mouse and human genetics. In humans, *SHH* dosage is critical for proper development, and loss of a single copy can result in the human condition holoprosencephaly (*69*, *70*). Furthermore, inappropriate activation of *Shh* expression (*67*, *71*, *72*) or Hh signaling (*73*–*75*) has been implicated in overproliferation phenotypes in diseases such as the Gorlin syndrome, basal cell carcinoma, and medulloblastoma. Therefore, *Shh* expression and Hh signaling require tight control in both development and homeostasis. This study’s demonstration of the critical role of Yap-mediated biophysical pathway in inducing and maintaining *Shh* expression in the ventral organizing centers identifies mechanotransduction as a critical upstream trigger of a cascade of integrated biophysical and biochemical signaling events in embryonic growth and patterning (Fig. 9D). The chordate notochord runs along almost the entire rostrocaudal body axis of amphioxus but terminates anteriorly in the region of the hindbrain of tunicates and vertebrates. As notochord is evolved to support the movement of animals with increased body sizes, by modification of a ventromedian muscle followed by the assembly of an axial complex supporting swimming in vertebrate ancestors, notochord formation is intrinsically subject to mechanoregulation. Integration of mechanotransduction in vertebrate notochord with the production of Shh that can form a signaling gradient to induce differentiation in the overlying CNS and the surrounding muscle and bony tissues allows the establishment of complex body plans of vertebrate animals.

The *Yap* CKO and *Yap/Taz* DKO mutant embryos exhibited interesting variation of phenotypic severity along the A-P axis. The lack of obvious morphological defects in the head is likely due to differential regulation of *Shh* expression along the A-P axis. At more rostral levels, *Shh* expression is likely also dependent on additional factors, as our results showed that Shh expression persisted after removal of *Yap/Taz*, albeit at weakened levels. However, activated Yap was sufficient to promote Shh expression in the embryonic brain, indicating Yap still plays critical roles in the rostral CNS development. In vivo reporter assays have led to the identification of several enhancers in the *Shh* gene that can direct reporter gene expression to discrete regions along the A-P axis of the ventral CNS in transgenic mice (*28*). In these findings, *Shh* expression is partially regulated by Yap/Tead in forebrain development through the *Shh* brain enhancer 1 (SBE1), and multiple factors are involved in regulating *Shh* expression via SBE (*28*, *76*). Furthermore, because the SFPE2 enhancer activity is dependent on the adjacent SBE1 activity (*28*), multiple TBSs on SBE1 and SFPE2 may have a synergistic effect on promoting *Shh* expression in the FP. Last, as transcription mediators of mechanotransduction, the contribution of Yap/Taz in regulating *Shh* expression may depend on the context of mechanotransduction. In the early limb bud, *Shh* expression was less dependent on Yap/Taz, possibly due to less morphogenetic changes in the limb bud compared to the notochord and the folding NT. In the adult mice, however, we found that in the subcutaneous mesenchymal progenitor cells, Yap is again both necessary and sufficient for the induction of ectopic *Shh* expression (*77*), suggesting that *Shh* induction in the adult mesenchymal cells might be more sensitive to Yap regulation.

It has been shown that removal of *Shh* from the notochord, not the FP, resulted in the loss of IVD and vertebral structures (*78*). Therefore, the most critical requirement of *Yap* for *Shh* expression appears to occur in the notochord, not the FP. Genetic or pharmacological activation of Hh signaling in the *Yap/Taz* DKO mutant embryos was sufficient to restore Shh expression in the NT, but not in the notochord, and notochord formation was not rescued. This is consistent with independent regulation of *FoxA2* expression in notochord and FP by the notochord enhancer (NE) and FP enhancer (FPE), respectively (*79*). While TBEs were found in the NE (*80*), they were not found in the FPE, which instead contains multiple Gli binding sites (*79*), and Hh signaling is required for minimal FPE activity (*58*). Previous studies showed that Tead1 and Tead2 are redundant regulators promoting *FoxA2* expression in the notochord, and Yap can activate NE of *FoxA2* in vitro (*64*, *80*). Here, we demonstrate that while *Yap* is strictly required to activate *FoxA2* and *Shh* expression in the notochord, they are not required for *Shh* and *FoxA2* expression in the FP when Hh signaling is activated. These findings indicate that strong Gli activation by Hh signaling can surpass the requirement of Yap for *Shh* expression in the FP, possibly due to strongly up-regulated *FoxA2* expression via FPE. Therefore, we think that in the notochord, the key target of Yap is *FoxA2*, but in FP, the key target of Yap is *Shh* (Fig. 9D). Consistently, no TBS has been found in the *FoxA2* FPE.

*Shh* expression in FP is regulated by FoxA2-dependent and FoxA2-independent mechanism through SFPE2 and SFPE1, respectively (*28*). The lack of TBSs in SFPE1, which does not drive *Shh* expression in the notochord (*28*), suggests that Yap regulates *Shh* expression in the FP through SFPE2 and depends on FoxA2. While Shh and FoxA2 can form a positive feedback loop in the FP, our results show that such feedback loop also requires Yap. In this regard, Yap uses a feedforward mechanism to activate robust *Shh* and *FoxA2* expression in the FP (Fig. 9D). Although Tead sites were not found in SFPE1, loss of *Shh* expression in the FP in the absence of Yap suggests that Yap may interact with SFPE1 via Tead-independent mechanism(s). While Yap plays an essential role to induce Shh expression, possibly by reading the mechanical information in the notochord and NT, we should point out that Yap also plays general roles of promoting cell proliferation and survival in early embryos, as revealed by the phenotypes of the *Yap^−/−^* mutants. Even with the conditional *Yap* deletion using the *ShhCre*, *Yap*, and *Shh* shows overlapping (FP) and distinct roles (notochord).

Conditional removal of *Yap/Taz* using *ShhCre* allowed clear demonstration of their previously unknown roles in notochord and FP formation by directly regulating *FoxA2* and *Shh* expression, respectively. Note that Yap is specifically activated in the notochord and FP. It is likely that comparing to maintain normal cell proliferation or survival in the entire embryos including the NT, higher Yap activities are required to drive *FoxA2* or *Shh* expression in the notochord or FP, respectively. Yap protein does not show a DV gradient similar to stress fiber distribution; rather, it is steeply up-regulated in the FP and notochord cells. It is possible that Yap up-regulation requires tissue stiffness above a certain threshold. In addition, the level of Yap in the ventral FP could also result from positive Yap-Shh feedback as we have recently identified in heterotopic ossification (*77*), which will sharpen the activation domain of both Yap/Taz and Shh expression. Note that Yap regulation of Shh expression is likely a generalizable mechanism that operates in distinct embryonic and adult tissues. We also found up-regulation of Yap and Ctgf in the dorsal NT (Fig. 2B), where Yap localization and function in the neural crest territory, a dorsal tissue at the time or NT closure, has been nicely reported (*49*). However, we did not observe increased tissue stiffness in the dorsal NT. While this could be a timing issue, we speculate that dorsal up-regulation of Yap may not be a result of increased tissue stiffness, rather it is directly controlled by the upstream Hippo kinase Mst1/2 and/or their targets, large tumor suppressor kinase 1/2 (Lats1/2) (*49*). NTDs are one of the most common birth defects in humans (*2*, *3*, *81*). Despite limited interventions of surgery corrections and folic acid supplementation during pregnancy, a significant fraction of NTDs remain unpreventable and/or difficult to treat (*6*). Our studies here suggest that Yap/Taz could be relevant targets to enhance weakened SHH signaling in NTDs.

## MATERIALS AND METHODS

### Study design

All mice were randomly grouped into mutant or treatment and control groups, and all measurements were performed in a blinded manner with respect to genotypes and treatment. Cohort sizes for in vivo experiments testing interventions were determined on the basis of an average variability of about 25% observed in previous animal cohorts or experimental groups, as well as a hypothesized change of one-half– or twofold due to the intervention. With these assumptions, sample sizes for sample group or cohorts of 4 would be 95% powered to detect this difference with an α of 0.05, while sample sizes with sample group or cohorts of 3 would be 90% powered to detect these differences with an α of 0.05. Thus, for each of the AFM and immunostaining studies, experimental arms were designed with an *n* of 3 or 4 in critical experiments and a minimum *n* of 3 in replication studies. Because the genetic or pharmacological intervention in the experiments did not cause detectible general health or survival issue, we included all animals without exclusion. For histologic data, a minimum of 20 cryosections from each animal subject were stained, and at least 4 to 6 stained sections were photographed and scored in a blinded fashion by two independent observers. Histologic measurements and qRT-PCR–based gene expression analyses were presented as the mean and SD of all animals in each group and reported without exclusion.

### Mice

All animal experiments were carried out according to protocols approved by the Institutional Animal Care and Use Committee at Harvard Medical School. Mouse lines are described in published literatures and purchased from the Jackson Laboratory: *ShhGFP-Cre* (Jackson Laboratory stock no. 005622), *Prrx1Cre* (Jackson Laboratory stock no. 005584), *Sox2CreER* (Jackson Laboratory stock no. 017593), *tdTMT^tg/+^*(Rosa26-TdTomato; Jackson Laboratory stock no. 007909), *Taz^f/f^/Yap^f/f^* (Jackson Laboratory stock no. 030532), conditional Yap^*^ or *Yap^gof^* (*Rosa26*^lox-stop-lox-rtTA/+^; *Col1a1*^*Teto*-YapS127A/+^) (*38*), *Ptch1^LacZ^* (*53*), and *Ctgf-GFP* (*38*). Timed mating of heterozygous intercrosses was performed to generate embryos of the indicated embryonic stage. The day in which a vaginal plug was confirmed was designated as E0.5. Mutant and transgenic embryos were processed in parallel with littermate controls. For the ShhCre;Yap^*^ embryos, Dox was administered to pregnant female mice at 0.2 mg/ml in drinking water starting at E8.5. For Sox2CreER;Yap^*^ embryos, Dox (same as above) and TM were administered at E7.5 (via intraperitoneal injection of the pregnant females (75 mg/kg of body weight).

### Immunofluorescence, confocal imaging, and analysis

Embryos were dissected in cold phosphate-buffered saline (PBS) and fixed in 4% paraformaldehyde for 2 hours. Then embryos were dehydrated through a gradient consisting of 10, 20, and 30% sucrose at 4°C and processed for cryostat sections. Cryosections were washed in PBS containing 0.1% Tween 20 (PBST) at room temperature, blocked in 10% donkey serum, and incubated with primary antibody at 4°C overnight. The sections were washed once in PBST and twice in PBS and then incubated with secondary antibodies at room temperature for 2 hours, followed by PBST washes. Stained sections were mounted in fluorescence mounting medium with 4′,6-diamidino-2-phenylindole (DAPI) (Millipore Sigma, D9542). All the antibodies are listed in table S1. The phalloidin staining of cryosections was performed by preincubating in PBS with 1% bovine serum albumin for 30 min, followed by incubating in 1:40 diluted (in PBS) phalloidin methanolic stock solution for 30 min at room temperature. The sections were washed in PBS before being mounted with DAPI. The images were captured using a ZEISS LSM 700 laser microscope. 2.5D view was performed by ZEN software and analyzed and quantified using ImageJ as described (*82*). Briefly, an area from the dorsal to the ventral of the NT was marked using ImageJ, which is a vector line segment covering the NT/neural plate and the notochord. The *x* axis represents distance, normalized to a scale of 0 to 1, from the dorsal domain (0) to the ventral domain (1.0).The *y* axis is the mean gray value that represents phalloidin immunofluorescence intensity.

### Skeletal preparation

Alcian Blue staining for cartilage and Alizarin Red staining for mineralized tissues were performed similarly as previously described (*83*). Specifically, E18.5 embryos were eviscerated, fixed in 95% ethanol overnight at room temperature, and then transferred into acetone overnight, followed by staining with Alizarin Red S (Sigma-Aldrich) and Alcian Blue (Sigma-Aldrich). Samples were cleared in 1% KOH and stored in 100% glycerol.

### AFM measurements of NT stiffness

Silicon nitride cantilever with a spring constant of 0.10 N/m and 20 ± 0.5 nm of radius silicon nitride particle attached to the tip (Asylum Research, model RC800PSA) was mounted on an Asylum SPM-2 (Asylum Research, Oxford Instruments) AFM. The embryos were embedded in O.C.T. immediately after dissection, and 35-μm cryosections were made and collected on Superfrost plus slides (Thermo Fisher Scientific, no. 48311-703). After immerged in PBS for 1 hour, samples were then mounted on the AFM *xy*-motorized stage for testing. The cantilever was calibrated, and the spring constant was determined according to the thermal noise method (*84*). Indentation measurements (triggering force: 1 nN) of the measured area were performed automatically and created a two-dimensional “stiffness map” of the area. Depending on the total sample size, each AFM mapping area was set at 14 μm by 14 μm (E8.5) and 20 μm by 20 μm (E9.5).

### Luciferase assay

Human embryonic kidney 293T cells (CRL-3216) obtained from American Type Culture Collection were cultured in Dulbecco’s modified Eagle’s medium (DMEM) supplemented with 10% fetal bovine serum (Sigma-Aldrich) and 1% penicillin-streptomycin (Gibco). To generate luciferase reporter constructs, HR-c and TBS-mutated HR-c DNAs were inserted into *pGL3* (Promega). Cotransfection of *pGL3* and *pRL-TK* (Promega) with *pCMV-YAP^5SA^* (Addgene, #27371) and/or *pCMV-Foxa2* (self-made) was performed using PEI (Polysciences) according to the manufacturer’s protocol. Luciferase activity was measured in triplicate using the Dual-Luciferase Reporter Assay System (Promega), and relative luciferase activity was calculated as the ratio of Firefly to Renilla luciferase activity.

### Data analysis

Tissues behave relatively elastic within the small strain ranges induced by AFM indentation, allowing to describe tissue mechanical properties, such as stiffness, as Young’s elastic modulus “*E*” in pascal (Pa) (*85*). First, force-displacement (*F*-*z*) curves were produced by translating cantilever deflection into force. *E* of the probed area surface was then calculated by fitting the contact part of the measured approach force curves to a standard Hertz model (*86*) using the equation shown below

$$E=\frac{3\;(1-v^{2})F}{4\;R^{\frac{1}{2}}\;\delta^{\frac{3}{2}}}$$

where *E* is the Young’s modulus, *v* is the Poisson’s ratio, *F* is force applied to the indenter (1 nN), *R* is the radius of the tip curvature (20 ± 5 nm), and epsilon (δ) is the indentation depth.

To quantify and visualize data from AFM indentation experiments, raw data files generated by Asylum software were loaded into MATLAB (MathWorks, Natick, MA) using an open-source script (www.mathworks.com/matlabcentral/fileexchange/80212-ardf-to-matlab). *E* values of each measured point were retrieved by this script. A heatmap was then generated using a customized MATLAB script with a convolution averaging feature.

### Collagen-alginate gel formation and explant culture

Collagen-alginate gel preparation for explant culture was performed similarly as previously described (*39*). Briefly, collagen type 1 (8 mg/ml) from rat tail (Corning) and 5% alginate (Sigma-Aldrich) were mixed at a 3:1 ratio on ice. Calcium carbonate solution (CaCO_3_; Sigma-Aldrich; 20 mM) was added to get more stable alginate gelation. Collagen solution was neutralized with 10× PBS (Corning) and 1 N NaOH (Sigma-Aldrich). Alginate lyase (Sigma-Aldrich, A1603) was used to soften the collagen-alginate gel. The stiff collagen-alginate gel was approximately 6.8 kPa and the soft one was 0.5 kPa.

Embryos were dissected and incubated in dispase (0.5 mg/ml; Sigma-Aldrich, D4693) in DMEM for 5 min as previously described (*23*). For E8.5 embryos, the intermediate neural plate was carefully dissected, and the adjacent mesoderm was excluded. The isolated neural tissues were then embedded with collagen-alginate gel, allowed for gel formation at 37°C for 1 hour, and then cultured in F12 culture media supplemented with N2 (Gibco, 17502048) and 1% heat-inactivated horse serum for 2 days. For drug treatment, E9.5 embryos were dissected and incubated in culture medium for 2 days. The ROCK inhibitor Y27632 (added for 24 hours at 15 μM concentration) and Cyt D (added for 24 hours at 5 μM concentration) were from Sigma-Aldrich.

### Whole-mount in situ hybridization

Whole-mount in situ hybridization was performed using digoxygenin-labeled antisense RNA according to standard protocols (*87*). Shh RNA probes have been described previously (*83*).

### Small-molecule treatment

SAG (MedChemExpress, HY-12848) was diluted in 10% dimethyl sulfoxide and then mixed with corn oil. SAG was injected intraperitoneally into pregnant females (20 mg/kg) at E8.5. Equivalent volumes of vehicle were injected to control animals.

### Quantitative real-time PCR

Total RNA from embryonic NT in the truck region (between the forelimbs and hind limbs) devoid of somite and endoderm was prepared using the TRIzol reagent (Life Technologies) or RNeasy Mini Kit (QIAGEN) according to the manufacturer’s protocols. cDNA was synthesized from total RNA (1 to 2 μg) using SuperScript II Reverse Transcriptase with random primers (Life Technologies). qRT-PCR was performed using the SYBR Select Master Mix on StepOnePlus thermal cycler from Applied Biosystems. Gene expression levels were always given relative to glyceraldehyde 3-phosphate dehydrogenase (Gapdh). The primer sequences of the genes are given in table S2.

### Statistical analysis

All data analyses in this study were carried out using GraphPad Prism 8 (GraphPad Software). Quantifications were done from at least three independent experimental groups. Statistical analysis between groups was performed by two-tailed Student’s *t* test to determine significance when only two groups were compared. One-way analysis of variance (ANOVA) with Tukey’s post hoc tests was used to compare differences between multiple groups. *P* values of less than 0.05 and 0.01 were considered significant. Error bars on all graphs are presented as the SD of the mean unless otherwise indicated.

## Notes

View/request a protocol for this paper from *Bio-protocol*.

## References

[1] T. Ramesh, S. V. Nagula, G. G. Tardieu, E. Saker, M. Shoja, M. Loukas, R. J. Oskouian, R. S. Tubbs, Update on the notochord including its embryology, molecular development, and pathology: A primer for the clinician. Cureus 9, e1137 (2017). 28480155 10.7759/cureus.1137 PMC5418029

[2] K. S. Au, A. Ashley-Koch, H. Northrup, Epidemiologic and genetic aspects of spina bifida and other neural tube defects. Dev. Disabil. Res. Rev. 16, 6–15 (2010). 20419766 10.1002/ddrr.93 PMC3053142

[3] D. M. Juriloff, M. J. Harris, Insights into the etiology of mammalian neural tube closure defects from developmental, genetic and evolutionary studies. J. Dev. Biol. 6, 22 (2018). 30134561 10.3390/jdb6030022 PMC6162505

[4] L. E. Mitchell, Epidemiology of neural tube defects. Am. J. Med. Genet. 135C, 88–94 (2005). 15800877 10.1002/ajmg.c.30057

[5] J. B. Wallingford, L. A. Niswander, G. M. Shaw, R. H. Finnell, The continuing challenge of understanding, preventing, and treating neural tube defects. Science 339, 1222002 (2013). 23449594 10.1126/science.1222002 PMC3677196

[6] N. D. Greene, A. J. Copp, Neural tube defects. Annu. Rev. Neurosci. 37, 221–242 (2014). 25032496 10.1146/annurev-neuro-062012-170354 PMC4486472

[7] H. J. Blom, G. M. Shaw, M. den Heijer, R. H. Finnell, Neural tube defects and folate: Case far from closed. Nat. Rev. Neurosci. 7, 724–731 (2006). 16924261 10.1038/nrn1986 PMC2970514

[8] M. Valet, E. D. Siggia, A. H. Brivanlou, Mechanical regulation of early vertebrate embryogenesis. Nat. Rev. Mol. Cell Biol. 23, 169–184 (2022). 34754086 10.1038/s41580-021-00424-z

[9] S. Piccolo, H. L. Sladitschek-Martens, M. Cordenonsi, Mechanosignaling in vertebrate development. Dev. Biol. 488, 54–67 (2022). 35580730 10.1016/j.ydbio.2022.05.005

[10] M. A. Wozniak, C. S. Chen, Mechanotransduction in development: A growing role for contractility. Nat. Rev. Mol. Cell Biol. 10, 34–43 (2009). 19197330 10.1038/nrm2592 PMC2952188

[11] S. Balmer, S. Nowotschin, A. K. Hadjantonakis, Notochord morphogenesis in mice: Current understanding & open questions. Dev. Dyn. 245, 547–557 (2016). 26845388 10.1002/dvdy.24392 PMC4844759

[12] M. Suzuki, H. Morita, N. Ueno, Molecular mechanisms of cell shape changes that contribute to vertebrate neural tube closure. Dev. Growth Differ. 54, 266–276 (2012). 22524600 10.1111/j.1440-169X.2012.01346.x

[13] J. M. Sawyer, J. R. Harrell, G. Shemer, J. Sullivan-Brown, M. Roh-Johnson, B. Goldstein, Apical constriction: A cell shape change that can drive morphogenesis. Dev. Biol. 341, 5–19 (2010). 19751720 10.1016/j.ydbio.2009.09.009 PMC2875788

[14] S. Dupont, L. Morsut, M. Aragona, E. Enzo, S. Giulitti, M. Cordenonsi, F. Zanconato, J. le Digabel, M. Forcato, S. Bicciato, N. Elvassore, S. Piccolo, Role of YAP/TAZ in mechanotransduction. Nature 474, 179–183 (2011). 21654799 10.1038/nature10137

[15] T. Panciera, L. Azzolin, M. Cordenonsi, S. Piccolo, Mechanobiology of YAP and TAZ in physiology and disease. Nat. Rev. Mol. Cell Biol. 18, 758–770 (2017). 28951564 10.1038/nrm.2017.87 PMC6192510

[16] Y. Zheng, D. Pan, The hippo signaling pathway in development and disease. Dev. Cell 50, 264–282 (2019). 31386861 10.1016/j.devcel.2019.06.003 PMC6748048

[17] B. Zhao, X. Wei, W. Li, R. S. Udan, Q. Yang, J. Kim, J. Xie, T. Ikenoue, J. Yu, L. Li, P. Zheng, K. Ye, A. Chinnaiyan, G. Halder, Z. C. Lai, K. L. Guan, Inactivation of YAP oncoprotein by the Hippo pathway is involved in cell contact inhibition and tissue growth control. Genes Dev. 21, 2747–2761 (2007). 17974916 10.1101/gad.1602907 PMC2045129

[18] F. D. Camargo, S. Gokhale, J. B. Johnnidis, D. Fu, G. W. Bell, R. Jaenisch, T. R. Brummelkamp, YAP1 increases organ size and expands undifferentiated progenitor cells. Curr. Biol. 17, 2054–2060 (2007). 17980593 10.1016/j.cub.2007.10.039

[19] Q. Y. Lei, H. Zhang, B. Zhao, Z. Y. Zha, F. Bai, X. H. Pei, S. Zhao, Y. Xiong, K. L. Guan, TAZ promotes cell proliferation and epithelial-mesenchymal transition and is inhibited by the hippo pathway. Mol. Cell. Biol. 28, 2426–2436 (2008). 18227151 10.1128/MCB.01874-07 PMC2268418

[20] M. Ota, H. Sasaki, Mammalian Tead proteins regulate cell proliferation and contact inhibition as transcriptional mediators of Hippo signaling. Development 135, 4059–4069 (2008). 19004856 10.1242/dev.027151

[21] K. Duning, D. Rosenbusch, M. A. Schlüter, Y. Tian, K. Kunzelmann, N. Meyer, U. Schulze, A. Markoff, H. Pavenstädt, T. Weide, Polycystin-2 activity is controlled by transcriptional coactivator with PDZ binding motif and PALS1-associated tight junction protein. J. Biol. Chem. 285, 33584–33588 (2010). 20833712 10.1074/jbc.C110.146381 PMC2962456

[22] E. M. Morin-Kensicki, B. N. Boone, M. Howell, J. R. Stonebraker, J. Teed, J. G. Alb, T. R. Magnuson, W. O'Neal, S. L. Milgram, Defects in yolk sac vasculogenesis, chorioallantoic fusion, and embryonic axis elongation in mice with targeted disruption of Yap65. Mol. Cell. Biol. 26, 77–87 (2006). 16354681 10.1128/MCB.26.1.77-87.2006 PMC1317614

[23] E. Marti, D. A. Bumcrot, R. Takada, A. P. McMahon, Requirement of 19K form of Sonic hedgehog for induction of distinct ventral cell types in CNS explants. Nature 375, 322–325 (1995). 7753196 10.1038/375322a0

[24] J. Ericson, J. Muhr, M. Placzek, T. Lints, T. M. Jessel, T. Edlund, Sonic hedgehog induces the differentiation of ventral forebrain neurons: A common signal for ventral patterning within the neural tube. Cell 81, 747–756 (1995). 7774016 10.1016/0092-8674(95)90536-7

[25] H. Roelink, J. A. Porter, C. Chiang, Y. Tanabe, D. T. Chang, P. A. Beachy, T. M. Jessell, Floor plate and motor neuron induction by different concentrations of the amino-terminal cleavage product of sonic hedgehog autoproteolysis. Cell 81, 445–455 (1995). 7736596 10.1016/0092-8674(95)90397-6

[26] J. A. Maier, Y. Lo, B. D. Harfe, Foxa1 and Foxa2 are required for formation of the intervertebral discs. PLOS ONE 8, e55528 (2013). 23383217 10.1371/journal.pone.0055528 PMC3561292

[27] H. Sasaki, B. L. Hogan, HNF-3β as a regulator of floor plate development. Cell 76, 103–115 (1994). 8287471 10.1016/0092-8674(94)90176-7

[28] D. J. Epstein, A. P. McMahon, A. L. Joyner, Regionalization of Sonic hedgehog transcription along the anteroposterior axis of the mouse central nervous system is regulated by Hnf3-dependent and -independent mechanisms. Development 126, 281–292 (1999). 9847242 10.1242/dev.126.2.281

[29] A. Ruiz i Altaba, T. M. Jessell, H. Roelink, Restrictions to floor plate induction by hedgehog and winged-helix genes in the neural tube of frog embryos. Mol. Cell. Neurosci. 6, 106–121 (1995). 7551564 10.1006/mcne.1995.1011

[30] Y. Jeong, D. J. Epstein, Distinct regulators of Shh transcription in the floor plate and notochord indicate separate origins for these tissues in the mouse node. Development 130, 3891–3902 (2003). 12835403 10.1242/dev.00590

[31] C. Chiang, Y. Litingtung, E. Lee, K. E. Young, J. L. Corden, H. Westphal, P. A. Beachy, Cyclopia and defective axial patterning in mice lacking Sonic hedgehog gene function. Nature 383, 407–413 (1996). 8837770 10.1038/383407a0

[32] M. P. Matise, D. J. Epstein, H. L. Park, K. A. Platt, A. L. Joyner, Gli2 is required for induction of floor plate and adjacent cells, but not most ventral neurons in the mouse central nervous system. Development 125, 2759–2770 (1998). 9655799 10.1242/dev.125.15.2759

[33] S. Walcott, S. X. Sun, A mechanical model of actin stress fiber formation and substrate elasticity sensing in adherent cells. Proc. Natl. Acad. Sci. U.S.A. 107, 7757–7762 (2010). 20385838 10.1073/pnas.0912739107 PMC2867880

[34] K. Hayakawa, H. Tatsumi, M. Sokabe, Actin filaments function as a tension sensor by tension-dependent binding of cofilin to the filament. J. Cell Biol. 195, 721–727 (2011). 22123860 10.1083/jcb.201102039 PMC3257564

[35] K. Burridge, E. S. Wittchen, The tension mounts: Stress fibers as force-generating mechanotransducers. J. Cell Biol. 200, 9–19 (2013). 23295347 10.1083/jcb.201210090 PMC3542796

[36] A. S. Shum, A. J. Copp, Regional differences in morphogenesis of the neuroepithelium suggest multiple mechanisms of spinal neurulation in the mouse. Anat. Embryol. (Berl) 194, 65–73 (1996). 8800424 10.1007/BF00196316

[37] T. Watanabe, H. Hosoya, S. Yonemura, Regulation of myosin II dynamics by phosphorylation and dephosphorylation of its light chain in epithelial cells. Mol. Biol. Cell 18, 605–616 (2007). 17151359 10.1091/mbc.E06-07-0590 PMC1783795

[38] D. Yimlamai, C. Christodoulou, G. G. Galli, K. Yanger, B. Pepe-Mooney, B. Gurung, K. Shrestha, P. Cahan, B. Z. Stanger, F. D. Camargo, Hippo pathway activity influences liver cell fate. Cell 157, 1324–1338 (2014). 24906150 10.1016/j.cell.2014.03.060 PMC4136468

[39] M. Jang, J. An, S. W. Oh, J. Y. Lim, J. Kim, J. K. Choi, J. H. Cheong, P. Kim, Matrix stiffness epigenetically regulates the oncogenic activation of the Yes-associated protein in gastric cancer. Nat. Biomed. Eng. 5, 114–123 (2021). 33288878 10.1038/s41551-020-00657-x

[40] L. V. Goodrich, R. L. Johnson, L. Milenkovic, J. A. McMahon, M. P. Scott, Conservation of the hedgehog/patched signaling pathway from flies to mice: Induction of a mouse patched gene by Hedgehog. Genes Dev. 10, 301–312 (1996). 8595881 10.1101/gad.10.3.301

[41] C. B. Bai, W. Auerbach, J. S. Lee, D. Stephen, A. L. Joyner, Gli2, but not Gli1, is required for initial Shh signaling and ectopic activation of the Shh pathway. Development 129, 4753–4761 (2002). 12361967 10.1242/dev.129.20.4753

[42] P. T. Chuang, A. P. McMahon, Vertebrate Hedgehog signalling modulated by induction of a Hedgehog-binding protein. Nature 397, 617–621 (1999). 10050855 10.1038/17611

[43] Y. Liu-Chittenden, B. Huang, J. S. Shim, Q. Chen, S. J. Lee, R. A. Anders, J. O. Liu, D. Pan, Genetic and pharmacological disruption of the TEAD-YAP complex suppresses the oncogenic activity of YAP. Genes Dev. 26, 1300–1305 (2012). 22677547 10.1101/gad.192856.112 PMC3387657

[44] E. Marti, R. Takada, D. A. Bumcrot, H. Sasaki, A. P. McMahon, Distribution of Sonic hedgehog peptides in the developing chick and mouse embryo. Development 121, 2537–2547 (1995). 7671817 10.1242/dev.121.8.2537

[45] B. D. Harfe, P. J. Scherz, S. Nissim, H. Tian, A. P. McMahon, C. J. Tabin, Evidence for an expansion-based temporal Shh gradient in specifying vertebrate digit identities. Cell 118, 517–528 (2004). 15315763 10.1016/j.cell.2004.07.024

[46] K. S. Choi, B. D. Harfe, Hedgehog signaling is required for formation of the notochord sheath and patterning of nuclei pulposi within the intervertebral discs. Proc. Natl. Acad. Sci. U.S.A. 108, 9484–9489 (2011). 21606373 10.1073/pnas.1007566108 PMC3111270

[47] P. T. Chuang, T. Kawcak, A. P. McMahon, Feedback control of mammalian Hedgehog signaling by the Hedgehog-binding protein, Hip1, modulates Fgf signaling during branching morphogenesis of the lung. Genes Dev. 17, 342–347 (2003). 12569124 10.1101/gad.1026303 PMC195990

[48] C. Lin, E. Yao, P. T. Chuang, A conserved MST1/2-YAP axis mediates Hippo signaling during lung growth. Dev. Biol. 403, 101–113 (2015). 25912685 10.1016/j.ydbio.2015.04.014 PMC4469623

[49] I. M. Martinez Traverso, J. D. Steimle, X. Zhao, J. Wang, J. F. Martin, LATS1/2 control TGFB-directed epithelial-to-mesenchymal transition in the murine dorsal cranial neuroepithelium through YAP regulation. Development 149, dev200860 (2022). 36125128 10.1242/dev.200860 PMC9587805

[50] B. K. Terry, S. Kim, The role of Hippo-YAP/TAZ signaling in brain development. Dev. Dyn. 251, 1644–1665 (2022). 35651313 10.1002/dvdy.504

[51] L. Madisen, T. A. Zwingman, S. M. Sunkin, S. W. Oh, H. A. Zariwala, H. Gu, L. L. Ng, R. D. Palmiter, M. J. Hawrylycz, A. R. Jones, E. S. Lein, H. Zeng, A robust and high-throughput Cre reporting and characterization system for the whole mouse brain. Nat. Neurosci. 13, 133–140 (2010). 20023653 10.1038/nn.2467 PMC2840225

[52] M. Logan, J. F. Martin, A. Nagy, C. Lobe, E. N. Olson, C. J. Tabin, Expression of Cre recombinase in the developing mouse limb bud driven by a Prxl enhancer. Genesis 33, 77–80 (2002). 12112875 10.1002/gene.10092

[53] L. V. Goodrich, L. Milenkovic, K. M. Higgins, M. P. Scott, Altered neural cell fates and medulloblastoma in mouse patched mutants. Science 277, 1109–1113 (1997). 9262482 10.1126/science.277.5329.1109

[54] J. K. Chen, J. Taipale, K. E. Young, T. Maiti, P. A. Beachy, Small molecule modulation of Smoothened activity. Proc. Natl. Acad. Sci. U.S.A. 99, 14071–14076 (2002). 12391318 10.1073/pnas.182542899 PMC137838

[55] M. Frank-Kamenetsky, X. M. Zhang, S. Bottega, O. Guicherit, H. Wichterle, H. Dudek, D. Bumcrot, F. Y. Wang, S. Jones, J. Shulok, L. L. Rubin, J. A. Porter, Small-molecule modulators of Hedgehog signaling: Identification and characterization of Smoothened agonists and antagonists. J. Biol. 1, 10 (2002). 12437772 10.1186/1475-4924-1-10 PMC137065

[56] J. Zhu, K. M. Kwan, S. Mackem, Putative oncogene Brachyury (T) is essential to specify cell fate but dispensable for notochord progenitor proliferation and EMT. Proc. Natl. Acad. Sci. U.S.A. 113, 3820–3825 (2016). 27006501 10.1073/pnas.1601252113 PMC4833266

[57] J. Briscoe, A. Pierani, T. M. Jessell, J. Ericson, A homeodomain protein code specifies progenitor cell identity and neuronal fate in the ventral neural tube. Cell 101, 435–445 (2000). 10830170 10.1016/s0092-8674(00)80853-3

[58] H. Sasaki, C. Hui, M. Nakafuku, H. Kondoh, A binding site for Gli proteins is essential for HNF-3β floor plate enhancer activity in transgenics and can respond to Shh in vitro. Development 124, 1313–1322 (1997). 9118802 10.1242/dev.124.7.1313

[59] J. Ericson, S. Thor, T. Edlund, T. M. Jessell, T. Yamada, Early stages of motor neuron differentiation revealed by expression of homeobox gene Islet-1. Science 256, 1555–1560 (1992). 1350865 10.1126/science.1350865

[60] K. Arnold, A. Sarkar, M. A. Yram, J. M. Polo, R. Bronson, S. Sengupta, M. Seandel, N. Geijsen, K. Hochedlinger, Sox2^+^ adult stem and progenitor cells are important for tissue regeneration and survival of mice. Cell Stem Cell 9, 317–329 (2011). 21982232 10.1016/j.stem.2011.09.001 PMC3538360

[61] S. L. Ang, J. Rossant, HNF-3 β is essential for node and notochord formation in mouse development. Cell 78, 561–574 (1994). 8069909 10.1016/0092-8674(94)90522-3

[62] D. C. Weinstein, A. Ruiz i Altaba, W. S. Chen, P. Hoodless, V. R. Prezioso, T. M. Jessell, J. E. Darnell Jr., The winged-helix transcription factor HNF-3β is required for notochord development in the mouse embryo. Cell 78, 575–588 (1994). 8069910 10.1016/0092-8674(94)90523-1

[63] S. Filosa, J. A. Rivera-Pérez, A. P. Gómez, A. Gansmuller, H. Sasaki, R. R. Behringer, S. L. Ang, Goosecoid and HNF-3β genetically interact to regulate neural tube patterning during mouse embryogenesis. Development 124, 2843–2854 (1997). 9226455 10.1242/dev.124.14.2843

[64] A. Sawada, H. Kiyonari, K. Ukita, N. Nishioka, Y. Imuta, H. Sasaki, Redundant roles of Tead1 and Tead2 in notochord development and the regulation of cell proliferation and survival. Mol. Cell. Biol. 28, 3177–3189 (2008). 18332127 10.1128/MCB.01759-07 PMC2423158

[65] Y. Tanabe, T. M. Jessell, Diversity and pattern in the developing spinal cord. Science 274, 1115–1123 (1996). 8895454 10.1126/science.274.5290.1115

[66] A. Lumsden, R. Krumlauf, Patterning the vertebrate neuraxis. Science 274, 1109–1115 (1996). 8895453 10.1126/science.274.5290.1109

[67] C. M. Fan, M. Tessier-Lavigne, Patterning of mammalian somites by surface ectoderm and notochord: Evidence for sclerotome induction by a hedgehog homolog. Cell 79, 1175–1186 (1994). 8001153 10.1016/0092-8674(94)90009-4

[68] R. L. Johnson, E. Laufer, R. D. Riddle, C. Tabin, Ectopic expression of Sonic hedgehog alters dorsal-ventral patterning of somites. Cell 79, 1165–1173 (1994). 8001152 10.1016/0092-8674(94)90008-6

[69] E. Belloni, M. Muenke, E. Roessler, G. Traverse, J. Siegel-Bartelt, A. Frumkin, H. F. Mitchell, H. Donis-Keller, C. Helms, A. V. Hing, H. H. Q. Heng, B. Koop, D. Martindale, J. M. Rommens, L. C. Tsui, S. W. Scherer, Identification of Sonic hedgehog as a candidate gene responsible for holoprosencephaly. Nat. Genet. 14, 353–356 (1996). 8896571 10.1038/ng1196-353

[70] E. Roessler, E. Belloni, K. Gaudenz, P. Jay, P. Berta, S. W. Scherer, L. C. Tsui, M. Muenke, Mutations in the human Sonic Hedgehog gene cause holoprosencephaly. Nat. Genet. 14, 357–360 (1996). 8896572 10.1038/ng1196-357

[71] Y. Echelard, D. J. Epstein, B. St-Jacques, L. Shen, J. Mohler, J. A. McMahon, A. P. McMahon, Sonic hedgehog, a member of a family of putative signaling molecules, is implicated in the regulation of CNS polarity. Cell 75, 1417–1430 (1993). 7916661 10.1016/0092-8674(93)90627-3

[72] A. E. Oro, K. M. Higgins, Z. Hu, J. M. Bonifas, E. H. Epstein Jr., M. P. Scott, Basal cell carcinomas in mice overexpressing sonic hedgehog. Science 276, 817–821 (1997). 9115210 10.1126/science.276.5313.817

[73] R. L. Johnson, A. L. Rothman, J. Xie, L. V. Goodrich, J. W. Bare, J. M. Bonifas, A. G. Quinn, R. M. Myers, D. R. Cox, E. H. Epstein Jr., M. P. Scott, Human homolog of patched, a candidate gene for the basal cell nevus syndrome. Science 272, 1668–1671 (1996). 8658145 10.1126/science.272.5268.1668

[74] N. Dahmane, J. Lee, P. Robins, P. Heller, A. Ruiz i Altaba, Activation of the transcription factor Gli1 and the Sonic hedgehog signalling pathway in skin tumours. Nature 389, 876–881 (1997). 9349822 10.1038/39918

[75] J. Xie, M. Murone, S. M. Luoh, A. Ryan, Q. Gu, C. Zhang, J. M. Bonifas, C. W. Lam, M. Hynes, A. Goddard, A. Rosenthal, E. H. Epstein Jr., F. J. de Sauvage, Activating smoothened mutations in sporadic basal-cell carcinoma. Nature 391, 90–92 (1998). 9422511 10.1038/34201

[76] Y. Yao, P. J. Minor, Y. T. Zhao, Y. Jeong, A. M. Pani, A. N. King, O. Symmons, L. Gan, W. V. Cardoso, F. Spitz, C. J. Lowe, D. J. Epstein, Cis-regulatory architecture of a brain signaling center predates the origin of chordates. Nat. Genet. 48, 575–580 (2016). 27064252 10.1038/ng.3542 PMC4848136

[77] Q. Cong, Y. Liu, T. Zhou, Y. Zhou, R. Xu, C. Cheng, H. S. Chung, M. Yan, H. Zhou, Z. Liao, B. Gao, G. A. Bocobo, T. A. Covington, H. J. Song, P. Su, P. B. Yu, Y. Yang, A self-amplifying loop of YAP and SHH drives formation and expansion of heterotopic ossification. Sci. Transl. Med. 13, eabb2233 (2021). 34162750 10.1126/scitranslmed.abb2233 PMC8638088

[78] K. S. Choi, C. Lee, B. D. Harfe, Sonic hedgehog in the notochord is sufficient for patterning of the intervertebral discs. Mech. Dev. 129, 255–262 (2012). 22841806 10.1016/j.mod.2012.07.003 PMC3478436

[79] H. Sasaki, B. L. Hogan, Enhancer analysis of the mouse HNF-3 β gene: Regulatory elements for node/notochord and floor plate are independent and consist of multiple sub-elements. Genes Cells 1, 59–72 (1996). 9078367 10.1046/j.1365-2443.1996.04004.x

[80] A. Sawada, Y. Nishizaki, H. Sato, Y. Yada, R. Nakayama, S. Yamamoto, N. Nishioka, H. Kondoh, H. Sasaki, Tead proteins activate the Foxa2 enhancer in the node in cooperation with a second factor. Development 132, 4719–4729 (2005). 16207754 10.1242/dev.02059

[81] A. J. Copp, N. D. Greene, Neural tube defects—Disorders of neurulation and related embryonic processes. Wiley Interdiscip. Rev. Dev. Biol. 2, 213–227 (2013). 24009034 10.1002/wdev.71 PMC4023228

[82] G. Nagamatsu, S. Shimamoto, N. Hamazaki, Y. Nishimura, K. Hayashi, Mechanical stress accompanied with nuclear rotation is involved in the dormant state of mouse oocytes. Sci. Adv. 5, eaav9960 (2019). 31249869 10.1126/sciadv.aav9960 PMC6594774

[83] Y. Yang, P. Guillot, Y. Boyd, M. F. Lyon, A. P. McMahon, Evidence that preaxial polydactyly in the Doublefoot mutant is due to ectopic Indian Hedgehog signaling. Development 125, 3123–3132 (1998). 9671585 10.1242/dev.125.16.3123

[84] E. K. Dimitriadis, F. Horkay, J. Maresca, B. Kachar, R. S. Chadwick, Determination of elastic moduli of thin layers of soft material using the atomic force microscope. Biophys. J. 82, 2798–2810 (2002). 11964265 10.1016/S0006-3495(02)75620-8 PMC1302067

[85] A. J. Engler, F. Rehfeldt, S. Sen, D. E. Discher, Microtissue elasticity: Measurements by atomic force microscopy and its influence on cell differentiation. Methods Cell Biol. 83, 521–545 (2007). 17613323 10.1016/S0091-679X(07)83022-6

[86] D. C. Lin, E. K. Dimitriadis, F. Horkay, Robust strategies for automated AFM force curve analysis—I. Non-adhesive indentation of soft, inhomogeneous materials. J. Biomech. Eng. 129, 430–440 (2007). 17536911 10.1115/1.2720924

[87] D. G. Wilkinson, M. A. Nieto, Detection of messenger RNA by in situ hybridization to tissue sections and whole mounts. Methods Enzymol. 225, 361–373 (1993). 8231863 10.1016/0076-6879(93)25025-w

