# Supplementary material for: Yap controls notochord formation and neural tube patterning by integrating mechanotransduction with FoxA2 and Shh expression
Source: Sci Adv. 2023 Jun 14;9(24):eadf6927. doi: 10.1126/sciadv.adf6927 (PMC10266736; doi:10.1126/sciadv.adf6927)
Supplement: Supplementary file 1 — Figs. S1 to S7 Table S1 and S2 [file sciadv.adf6927_sm.pdf]

Science Advances
AAAS

Supplementary Materials for

Yap controls notochord formation and neural tube patterning by integrating mechanotransduction with *FoxA2* and *Shh* expression

Caiqi Cheng *et al.*

Corresponding author: Yingzi Yang, yingzi_yang@hsdm.harvard.edu

Sci. Adv. **9**, eadf6927 (2023)
DOI: 10.1126/sciadv.adf6927

This PDF file includes:

Figs. S1 to S7
Tables S1 and S2

The Supplementary materials contain 7 figures and 2 tables.

Supplementary Figure 1-7

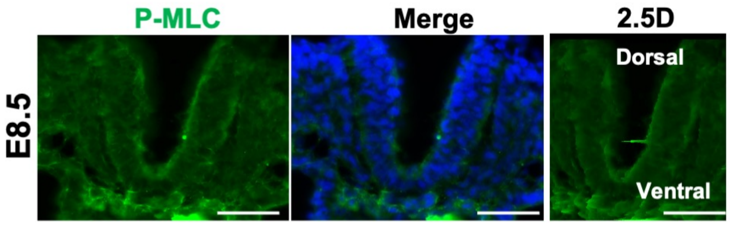

Figure S1. Representative immunofluorescent images of Phospho-Myosin Light Chain (P-MLC) and 2.5D reconstruction of laser scanning image stacks of P-MLC in E8.5 neural tube. Scale bar: 50 µm. (mean ± SD; n = 3 biological replicates).

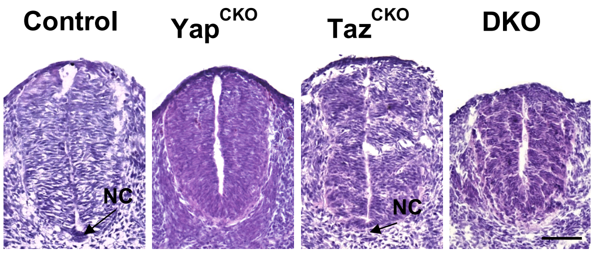

Figure S2. Hematoxylin and eosin (H&E) staining of neural tube in indicated groups at E10.5. Black arrow indicated notochord. Scale bar: 100 µm. not: notochord. (mean ± SD; n = 3 biological replicates).

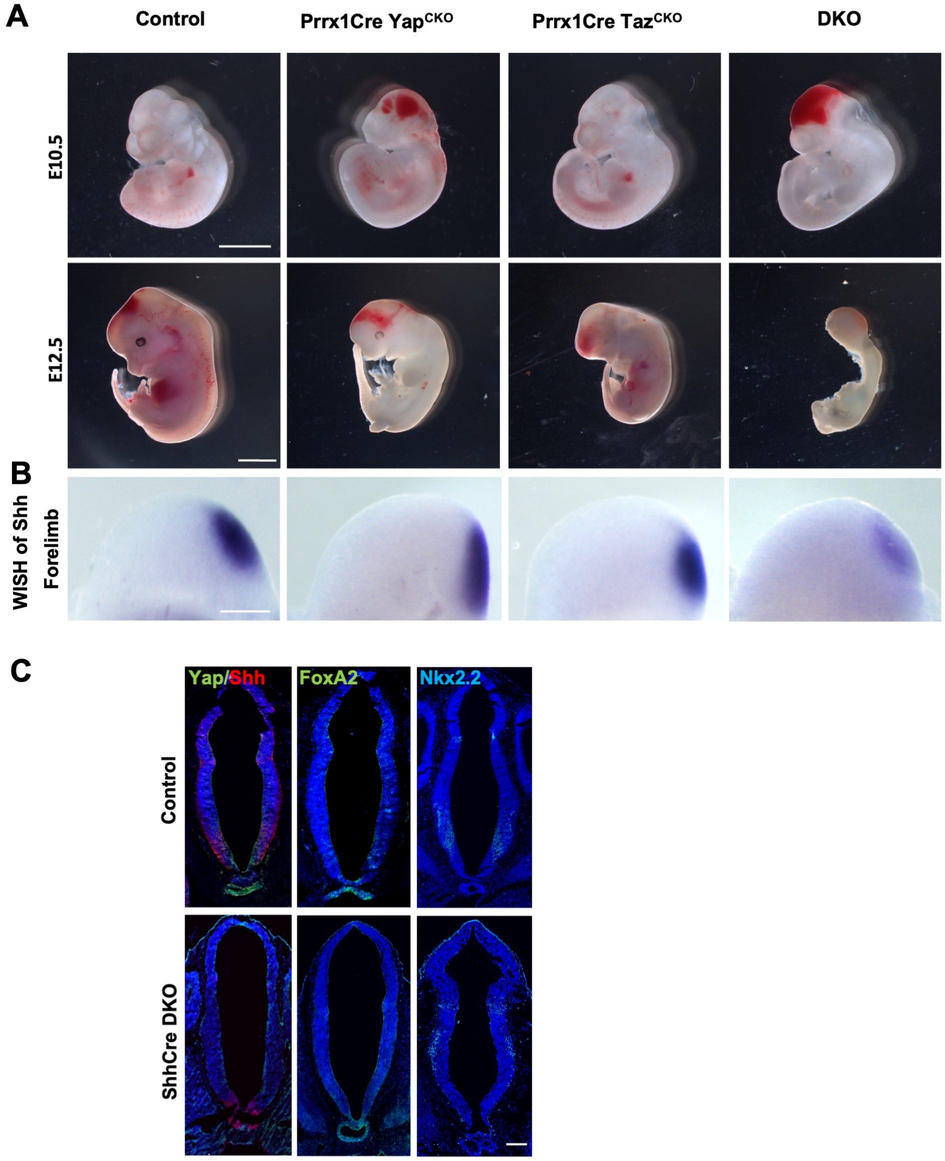

Figure S3. (A) Representative images of the E10.5 and E12.5 embryos of the indicated genotypes. Scale bar: 1mm. (B) Whole mount in situ hybridization for *Shh* expression in the forelimb bud of E10.5 embryos of indicated genotypes. The lower panel showed the forelimb buds. Scale bar: 250 µm. (C) Representative immunofluorescent images of Yap, Shh, FoxA2, Nkx2.2 at E10.5 in the midbrain of indicated genotypes. Scale bar: 200 µm. (mean ± SD; n = 3 biological replicates).

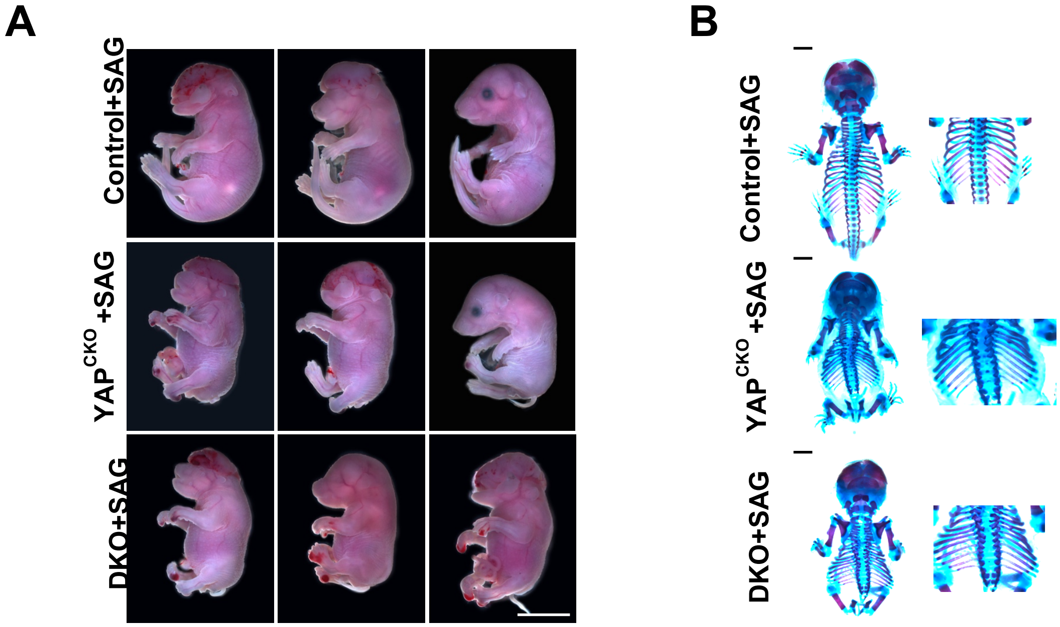

Figure S4. (A) Bright field images of the indicated E18.5 embryos with SAG treatment. Scale bar: 1 cm. (B) Whole mount images of E18.5 mouse embryos of indicated genotypes with SAG treatment after alizarin red and alcian blue staining. Dorsal view of ribcages is shown on the right panel. Scale bars: 1 mm.

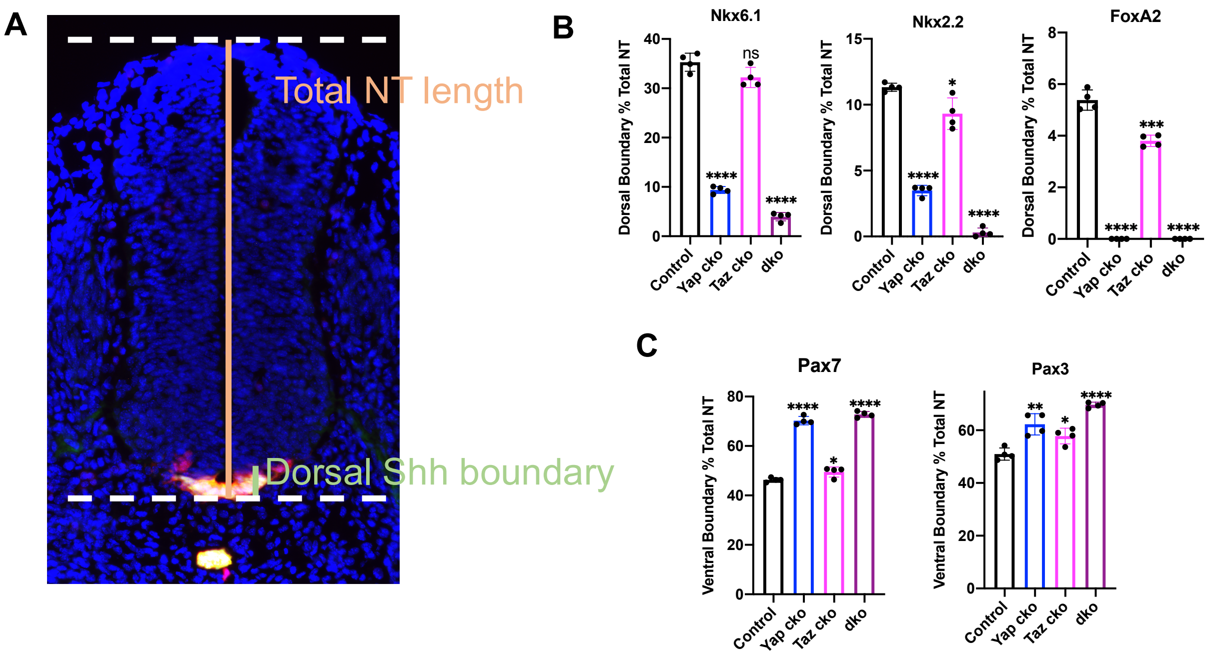

Figure S5. (A) schematics of the quantification of gene expression domains along the DV axis of the NT. Shh expression is indicated as an example. For the ventral gene (i.e., Shh) expression, the ratio of dorsal boundary length (ventral bottom to the dorsal boundary) vs the total NT length was calculated. For the dorsal gene (i.e., Pax3 and Pax7), the ratio of ventral boundary length (dorsal top to the ventral boundary) vs the total NT length was calculated. (B) Quantitation of dorsal boundary of Nkx6.1, Nkx2.2 and FoxA2 expression as a percentage of total D-V lumen length. (C) Quantitation of ventral boundary of Pax3 and Pax7 expression as a percentage of total D-V lumen length. (mean ± SD; n = 3 biological replicates). *P < 0.05, **P < 0.01, ***P < 0.001, ****P < 0.0001, ns: no significant difference one-way ANOVA followed by Tukey's multiple comparisons tests.

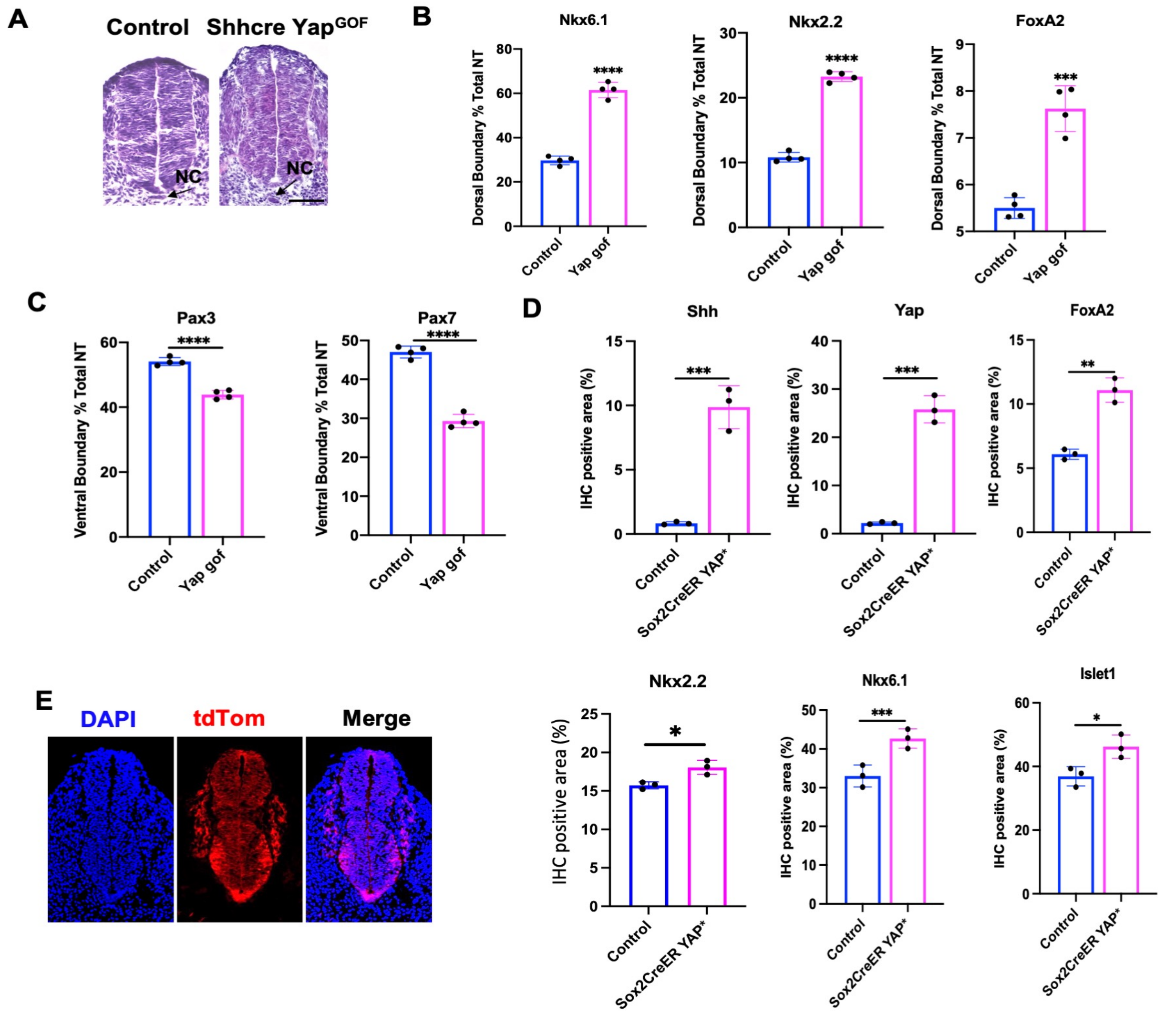

Figure S6. (A) Hematoxylin and eosin (H&E) staining of neural tube in control and *ShhCre*;*Yap** embryos at E10.5. Black arrows indicate notochord. Scale bar: 100 μm. NC: notochord. (B) Quantitation of dorsal boundary of Nkx6.1, Nkx2.2 and FoxA2 gene expression as a percentage of total D-V lumen length. (C) Quantitation of ventral boundary of Pax3 and Pax7 gene expression as a percentage of total D-V lumen length. (mean ± SD; n = 3 biological replicates). ****P < 0.0001 one-way ANOVA followed by Tukey's multiple comparisons tests. (D) Quantification of Shh, Yap, FoxA2, Nkx2.2, Nkx6.1, and Islet1 positive area at E10.5 in neural tube of indicated genotypes. (mean ± SD; N = 3 biological replicates). *P < 0.05, **P < 0.01, ***P < 0.001, ****P < 0.0001 one-way ANOVA followed by Tukey's multiple comparisons tests. (E) Sox2-TdTom positive cells in trunk cross sections of SoxCreER; Rosa26-tdTom embryos at E10.5. TM was injected at E7.5. Scale bar: 100 μm.

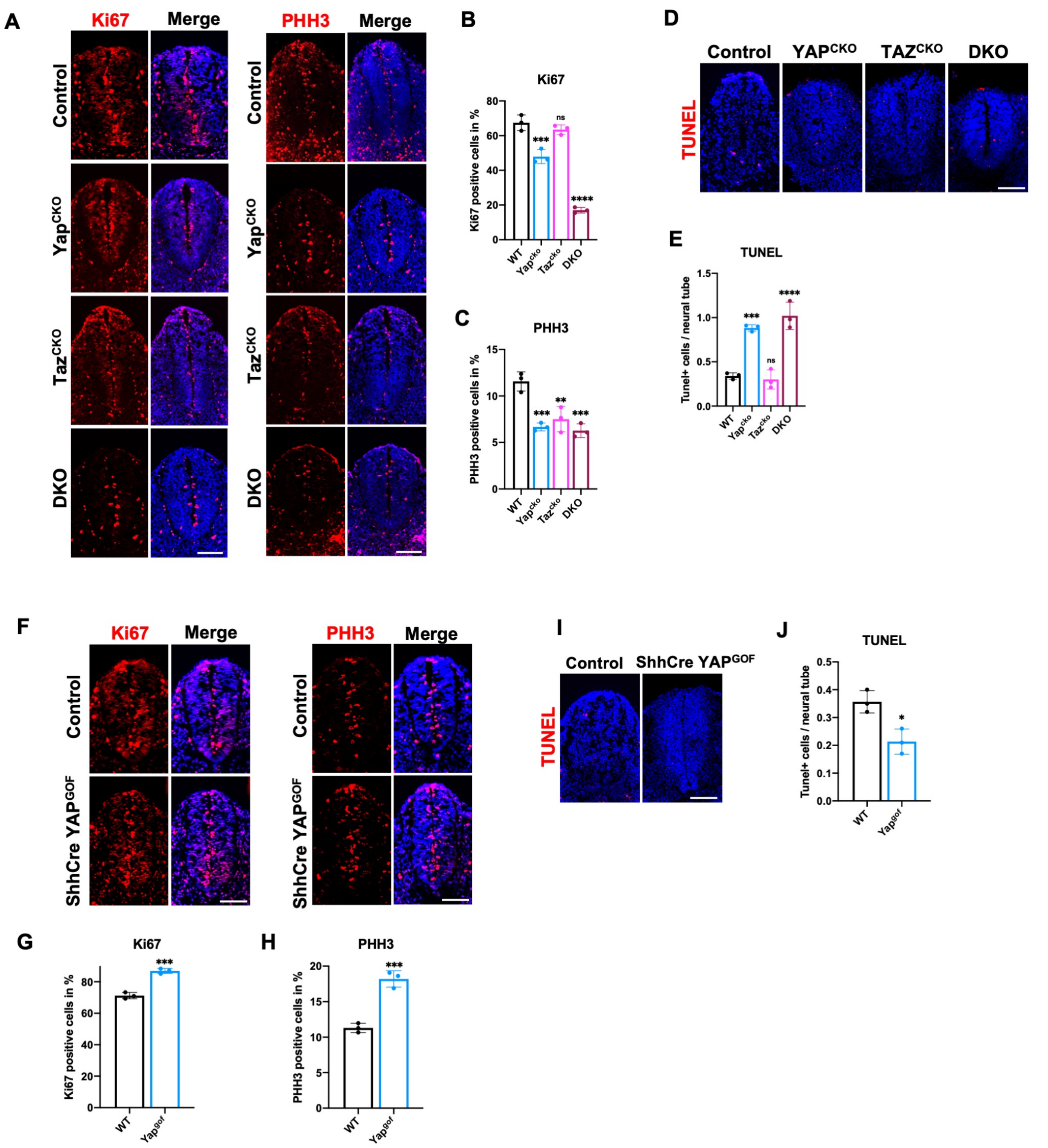

Figure S7. (A) Representative immunofluorescent images of Ki67 and PHH3 in neural tube of E10.5 embryos with indicated genotypes. Scale bar: 100 μm. (B, C) Bar graph represents percentage of Ki67$^+$ (B) and PHH3$^+$ (C) cells in the neural tube of E10.5 embryos of the indicated groups. (D) TUNEL staining of neural tube from indicated E10.5 embryos. (E) Quantification of TUNEL positive cells in the neural tube of indicated embryos. (F) Representative immunofluorescent images of Ki67 and PHH3 at E10.5 in the neural tube of indicated genotypes. (G, H) Bar graph represents percentage of Ki67$^+$ (G) and PHH3$^+$ (H) cells in the neural tube of indicated groups. (I) TUNEL staining of neural tube from indicated E10.5 embryos. (J) Quantification of TUNEL positive cells in the neural tube of indicated E10.5 embryos. Scale bars:

100 µm. (mean ± SD; n = 3 biological replicates). *P < 0.05, **P < 0.01, ***P < 0.001, ****P < 0.0001, ns: no significant difference one-way ANOVA followed by Tukey's multiple comparisons tests.

Table S1: Antibodies

**Reagent type (species) or resource**	**Designation**	**Supplier**	**Identifiers**
Antibody	SHH<br>Mouse mAb	Developmental Studies Hybridoma Bank	Cat# 5E1<br>RRID:AB_528466
Antibody	Anti-YAP (Rabbit monoclonal)	Cell Signalling Technology	Cat# 14074S,<br>RRID:AB_2650491
Antibody	Anti-GFP(Goat polyclonal)	Abcam	ab6673<br>RRID:AB_305643
Antibody	Nkx6.1<br>Mouse mAb	Developmental Studies Hybridoma Bank	Cat# F55A10<br>RRID:AB_532378
Antibody	Nkx2.2<br>Mouse mAb	Developmental Studies Hybridoma Bank	Cat#74.5A5<br>RRID:AB_531794
Antibody	FoxA2<br>Mouse mAb	Developmental Studies Hybridoma Bank	Cat#4C7,<br>RRID:AB_528255
Antibody	Pax3<br>Mouse mAb	Developmental Studies Hybridoma Bank	Cat#PAX3,<br>RRID:AB_528426
Antibody	Pax7<br>Mouse mAb	Developmental Studies Hybridoma Bank	Cat#Pax7,<br>RRID:AB_528428
Antibody	Brachyury<br>Polyclonal Goat Ab	R&D Systems	Cat#AF2085,<br>RRID:AB_2200235
Antibody	Ki67 Rabbit Polyclonal Ab	Thermo Fisher Scientific	Cat#PA5-19462,<br>RRID:AB_10981523
Antibody	Phospho-Histone H3 (Ser10) Rabbit Ab	Cell signaling Technology	Cat#9701<br>RRID:AB_331535
Antibody	PLMC Mouse mAb	Cell signaling Technology	Cat#3675<br>RRID:AB_2250969
Antibody	Alexa Fluor™ 647 Phalloidin	Thermo Fisher	Cat#A22287<br>RRID:AB_2620155
Antibody	Alexa Fluor 488 donkey anti-mouse (polyclonal)	Santa Cruz	Cat#sc-362258,<br>RRID:AB_11014318)
Antibody	Alexa Fluor 488 donkey	Life Technologies	Cat# A-21202,<br>RRID:AB_141607

	anti-rabbit (polyclonal)		
Antibody	Alexa Fluor 647 donkey anti-mouse (polyclonal)	Santa Cruz	Cat#sc-362288, RRID:AB_10988592
Antibody	Alexa Fluor 488 donkey anti-goat (polyclonal)	Life Technologies	Cat# A-21206, RRID:AB_2535792

Table S2: Sequences of qRT-PCR Primers

**qRT-PCR primers (mouse)**	**Forward 5'-3'**	**Reverse 5'-3'**
Gapdh	GCCTTCCGTGTTCCTACCC	TGCCTGCTTCACCACCTTC
Shh	GATGACTCAGAGGTGCAAAGACAA	TGGTTCATCACAGAGATGGCC
Cyr61	GCTCAGTCAGAAGGCAGACC	GTTCTTGGGGACACAGAGGA
Ctgf	CTGCCTACCGACTGGAAGAC	CATTGGTAACTCGGGTGGAG
Gli	GAAAGTCCTATTCACGCCTTGA	CAACCTTCTTGCTCACACATGTAAG
Ptch1	CTCTGGAGCAGATTTCCAAGG	TGCCGCAGTTCTTTTGAATG
Hhip	GGGAAAAACAGGTCATCAGC	ATCCACCAACCAAAGGGC
